# D-Light on promoters: a client-server system for the analysis and visualization of cis-regulatory elements

**DOI:** 10.1186/1471-2105-14-140

**Published:** 2013-04-24

**Authors:** Josef Laimer, Clemens J Zuzan, Tobias Ehrenberger, Monika Freudenberger, Simone Gschwandtner, Carina Lebherz, Peter Lackner

**Affiliations:** 1 Department of Molecular Biology, University of Salzburg, Hellbrunnerstr. 34, 5020 Salzburg, Austria; 2 Upper Austria University of Applied Sciences, Softwarepark 11, 4232 Hagenberg, Austria

## Abstract

**Background:**

The binding of transcription factors to DNA plays an essential role in the regulation of gene expression. Numerous experiments elucidated binding sequences which subsequently have been used to derive statistical models for predicting potential transcription factor binding sites (TFBS). The rapidly increasing number of genome sequence data requires sophisticated computational approaches to manage and query experimental and predicted TFBS data in the context of other epigenetic factors and across different organisms.

**Results:**

We have developed *D-Light*, a novel client-server software package to store and query large amounts of TFBS data for any number of genomes. Users can add small-scale data to the server database and query them in a large scale, genome-wide promoter context. The client is implemented in Java and provides simple graphical user interfaces and data visualization. Here we also performed a statistical analysis showing what a user can expect for certain parameter settings and we illustrate the usage of *D-Light* with the help of a microarray data set.

**Conclusions:**

*D-Light* is an easy to use software tool to integrate, store and query annotation data for promoters. A public *D-Light* server, the client and server software for local installation and the source code under GNU GPL license are available at http://biwww.che.sbg.ac.at/dlight.

## Background

The specific transcription of genes is largely controlled by the interplay of transcription factors (TFs) attached to their specific binding sites (TFBSs). It is commonly accepted that for higher organisms the concurrent binding of two or more TFs is required to change the transcriptional state of a gene. In addition, the evolutionary conservation of such binding pattern is assumed – although differences are expected [1].

A number of computational tools have been developed to process experimental data for subsequent prediction of potential TFBSs and affected pathways. Experimentally determined binding regions are sequenced and compared for common patterns by elaborate statistical methods as e.g. implemented in MEME [2] or WEEDER [3]. The obtained binding site data are collected in databases such as JASPAR [4] or TRANSFAC [5]. Finally, with different prediction methods [6-10] occasionally incorporating homologous genes [11,12] a huge amount of data for potential binding sites can be generated.

Currently several software packages or web servers are available to deal with these data. In general the different approaches are restricted to certain aspects or limited in the amount of data they are able to handle. For example, some tools are restricted to a only few genes [13,14] and just very few implementations utilize information from orthologous genes [15-18]. Other methods require additional experimental data such as expression levels [19]. Moreover the servers are not always easy to use or cannot be complemented with user data. To our knowledge, no service is available for performing combinatorial queries on a genome wide level with concurrent inference of orthologous genes. Finally, only very few tools are freely available for local installation.

Our client-server based software, *D-Light*, provides a new tool which aims to overcome most of these limitation. (i) *D-Light* allows for combinatorial searches within or between different species on a genome wide scale. (ii) The software provides a simple JAVA-based graphical user interface (GUI) available as browser applet, Java Web Start application or as stand-alone JAVA application. (iii) Users can add new promoter sequences, positional frequency matrices (PFMs) representing the TFs or generic annotations for their subsequent usage in the combinatorial queries. (iv) A user management enables privacy. (v) Client and server are open source software and can be installed locally. We also provide a public *D-Light* server pre-filled with data from human, mouse and rat.

Below we first describe the technical concepts and their implementation. We then use *D-Light* to determine *on average* useful promoter sizes and score cutoff values. We finally demonstrate the relevance of *D-Light* on a biological example.

## Implementation

**Figure 1:**
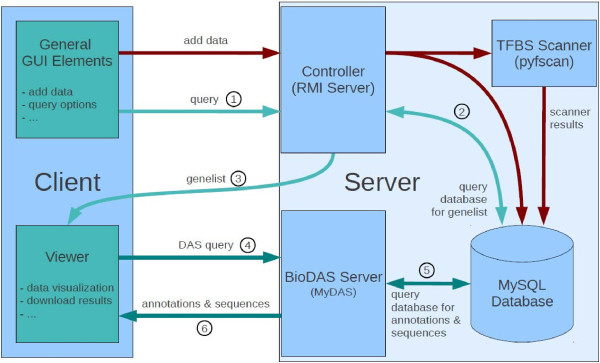
**Block diagram of the *D-Light *client-server system.** Green arrows represent the data flow on user queries, red arrows the data flow when a user uploads new data via the client GUI. Once a gene hit list is provided to the viewer, data are requested gene-wise via the DAS protocoll.

We first discuss design principles of the *D-Light* system regarding datasets, access control and data retrieval. Then we describe server and client characteristics. A scheme of software components and data flow is shown in Figure 1. The implementation of *D-Light* solely employs open source software.

### Design principles

*D-Light* implements a gene centered concept for data storage and access. The promoter is seen as a continuous region on the genomic DNA sequence which is associated with a certain gene and labeled with an accession code. Users added sequence data, however, may comprise any piece of genomic DNA labeled with a unique accession code. *D-Light* supports multiple genomes defined during server setup. Genomic coordinates provided for a certain promoter allow linkage to external genome browsers.

The second type of data stored in *D-Light* are features, which are in general binding sites of a certain transcription factor. User-added data may contain any type of features, with or without an associated score. If the user-added features are TFs with an associated PFM, an integrated prediction method allows the assignment of the TFs to all currently stored promoters.

All user-added data, namely promoters, features and the annotated locations thereof are private. Default data calculated during the *D-Light* server setup are accessible to all users. A certain user thus queries the union of default and his private data.

Inquiries to the server for the occurrences of certain features (or combinations thereof) are performed in two steps. First a list of promoters is generated, where at least one hit appears. Only during visualization of a certain promoter, all hits on that promoter are calculated (see Figure 1). This optimizes the response time for the interactive database queries. Complete hit lists are generated on request and can be downloaded in text format (csv format), which then requires more time.

### Server

All data are stored in a MySQL relational database. The database schema is optimized for speed rather than for space requirement. Stored procedures additionally speed up complex queries.

The server setup is controlled by an XML formatted setup file and performed with Python scripts. By default, the scripts download data from NCBI, the JASPAR ftp server and our web site. If *D-Light* should use different data sources or prediction tools the setup scripts can be easily modified. Related comments are included in the corresponding scripts.

The controller is written in Java to utilize RMI (Remote Method Invocation) for the client. Annotation data transfer is captured by the BioDAS protocol using the Java based MyDas server [20] (see also Figure 1).

### Client

The client is written in Java and communicates with the server via RMI. The GUI is based on AWT/Swing. The annotation viewer uses the GenoViz toolkit [21]. The client provides multiple query instances. The data required to visualize certain queries are stored in distinct objects which subsequently allows to change quickly between views.

All data visualized in lists, such as genes or features, are equipped with a filter which dynamically displays only records matching a regular expression style criterion.

### Sequential data flow on user queries

The numbers 1 to 6 in the flowchart (Figure 1) reflect the sequential data flow during a typical user query to display annotations for a set of genes. (1) A query string is passed to the controller, which translates it to SQL queries. (2) The SQL query results in a list of matching genes which is passed to the client (3). When the user selects a gene, the BioDAS server is contacted (4). Then, the BioDAS server formulates a SQL query (5) and returns the data via a DAS-xml envelop (6).

### TFBS prediction

The built-in binding site scanner, *pyfscan*, is written in C++ and implemented Python as extension. A stand-alone version of the TFBS scanner for usage independently of *D-Light* is included in the server package. By default, *pyfscan* calculates log-likelihood ratio scores with uniform background distribution *b*_*A*_ = *b*_*C*_ = *b*_*G*_ = *b*_*T*_ = 0.25:

$$S=\overset{L}{\underset{i=1}{\sum }}\ln\frac{f_{a,i}}{b_{a}}$$

where *i* is the *i*^*th *^column in the PFM, *a* denotes the current base given by the respective DNA sequence, *f*_*a*,*i *_is relative frequency of base *a *in column *i *and *L *is the length of the PFM.

The scores are converted into p-values using the method of Staden [22]. Raw scores are converted to normalized scores ranging from 0 to 100, where 100 corresponds to the maximum reachable score of a given PFM.

### Retrieving promoter data

Promoter data are extracted from UCSC chromosome files. Currently we only provide promoter data for genomes with assigned RefSeq IDs. Since *D-Light* implements a gene-focused concept, a certain RefSeq ID may only appear once in the promoter set. In practice, UCSC assigns some RefSeq ID to several chromosomal locations. If this is the case, we sort by chromosome name and take the first one. This way, the assignments in normal chromosomes are preferred compared to “random” or “unknown” entities, e.g. chr1 is preferred to chr1_random. In hg19, multiply used IDs affects 3% of all IDs. In many cases these are non coding RNAs or microRNAs.

The chromosomal location information is taken from the respective refGene tables. Following UCSCs strategy we extract ±1 kB, ±2 kB and ±10 kB regions relative to the proposed TSS. In contrast to UCSCs strategy for providing promoter fasta files we also include sequences with unknown TSS. In this case, the CSS (coding sequence start) will be known. In many cases the TSS and CSS are within a few hundred bases, and the extracted promoters are likely to include an active TSS (see statistical analysis).

For cross-genome search homology information is required. We use the homology relations defined by the gene product rather than by genomic alignments. The homologous groups are derived from NCBI HomoloGene database [23] and transferred to the MySQL database during the *D-Light* server setup.

### Default settings of the server

**Figure 2:**
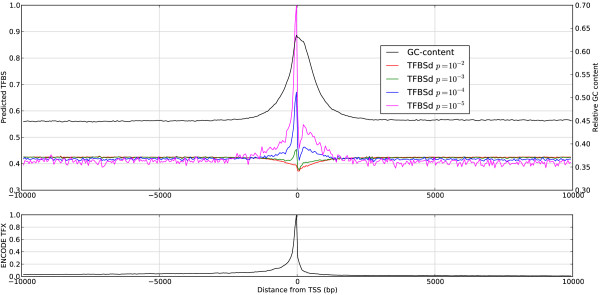
**GC-content, TFBS frequencies at various p-value cutoffs and ENCODE Txn occurrences.** Data are collected in bins of 50 bases from a non-redundant human promoter set consisting of approx. 23.000 ±10 kB sequences. Upper panel: For high cutoff values such as *p* = 10^−2^ (red line) the TFBS density (TFBSd) curve approximately mirrors the GC content (right axis). Lower p-value cutoffs result in curves with a sharp peak around the TSS. Lower panel: The distribution of ENCODE Txn peak centers. The left y-axes are scaled to one according to the maximum peak.

The space an time requirements of *D-Light* depend essentially on the promoter sizes, the size and composition of the PFM database and the p-value cutoff of the prediction method. We aimed to find parameter settings which are useful for an “average” gene. Considering the average density of predicted TFBS and average GC content (Figure 2), we decided to use ±2 kB promoter sequences by default. We also include genes with unknown TSS (see also section Results and discussion). Our defaults include the promoters for human, mouse and rat. There are, however, 25 genomes with RefSeq associations available (e.g. D. melanogaster or C. elegans) and provided for download on our web server.

In accordance with FIMO [24], which we used as reference TFBS prediction tool during our software development, the p-value cutoff is set to 10^−4^. By default, the JASPAR CORE Vertebrata set is enabled in *D-Light*, resulting in 116 usable PFMs which can achieve scores with a p-value smaller than 10^−4^.

## Results and discussion

We first show a statistical analysis of the promoters from the human genome regarding the distribution of predicted TFBS with respect to different parameters. Then we perform a case study applying *D-Light* to a microarray experiment based gene sets from a cell cycle study.

### Statistics on promoters

From the hg19 we created ±10 kB promoters set with annotated TSS consisting of approximately 23,000 sequences. We then predicted TFBS for the JASPAR CORE Vertebrata PFMs using the log-likelihood ratio score with different p-value cutoffs.

We first plotted the frequencies of predicted binding sites the GC content of the promoter sequences in intervals of 50 bases (Figure 2, upper panel). With a loose p-value cutoff (<0.01) the hits are almost equally distributed but with less hits around the TSS.

The average GC content of the applied PFMs is 47.4%. Therefore less hits appear in the GC reach regions around the TSS on average. The negative peak is rather small compared to the ground level. The PFMs which remain at lower p-value cutoffs only have a marginally higher GC content (49% for PFMs which reach p-values below 10^−5^). The positive peaks which appear at more stringent p-value cutoffs presumably appear because of the increasing number of real binding sites.

This result is supported by a comparison to ENCODE [25] data. We extracted the Txn Factor ChIP track data from the UCSC database and calculated the distance of the peak centers to the nearest TSS of RefSeq genes and plotted the relative frequencies of occurrence again with bin width of 50 bases (Figure 2, lower panel). The shape of the distribution is very similar to the curve of the predicted TFBS at p-value cutoff < 10^4^ and < 10^5^ respectively. Note that the base level of predicted TFBS is much higher than the base level of experimentally determined TFBS.

The peaks of all TFBS curves lie within two kilo-bases upstream the TSS and 1.5 kilo-bases downstream. Therefore we consider at least a ±2 kB region in D-Light.

Unfortunately, for many genes the TSS is not known. However, when the gene is coding for a protein, in general the start of the coding sequence (CSS) is known and annotated. We were interested in the distribution of the distance between the proposed TSS and CSS, in case that both entities are annotated. The distance varies between 1 base and a 1.9 mega bases. The quantiles are 114 (0.25-quartile), 373 (median), and 4280.25 (0.75-quartile). Therefore, in the majority of these cases the TSS is in close proximity to the CSS. In the human genome there are about 4000 genes with RefSeq association annotated with a CSS but not with a TSS. These genes are neglected by the UCSC rules for creating promoter sequences. We subsequently include such genes and use the CSS as anchor for defining the “up- and downstream” region, as only a fraction of those genes will have the TSS outside the proposed promoter region.

**Figure 3:**
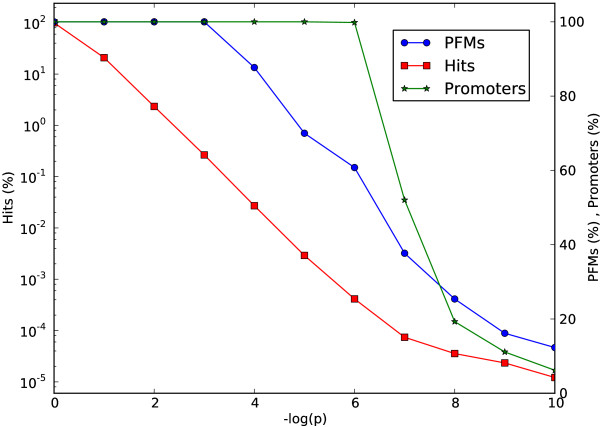
**Number of PFMs, hits and promoters for different p-value cutoffs.** The number of hits (red), number of affected promoters (green) and number of hit producing PMFs (blue) depend on the p-value cutoff. Note that the percentage of hits are plotted on the logarithmic scale (right side). The 100% baseline corresponds to the values obtained without any P-value cutoff, i.e. all possible positions for a hit, all promoters and all PFMs respectively.

Of significant importance for studying gene regulation on the basis of predicted TFBS’s is the balance between statistical significance of the hits and potentially false hits. Different authors use different p-value cutoffs for their methods to predict TFBS, commonly between 10^−4^ and 10^−6^. We therefore investigated the reachable significance of the 130 JASPAR CORE Vertebrata PFMs using our built-in prediction method. In Figure 3 we plotted the percentage of matrices able to achieve a certain p-value, given a uniform background distribution. With a p-value cutoff of 10^−4^ 13 PFMs can not obtain hits, namely GATA2, Prrx2, ETS1, NFE2L1::MafG, ARID3A, Arnt, Arnt::Ahr, GATA3, MZF1_1-4, NFIC, Pdx1, SOX10, YY1, ZEB1, ZNF354C, and Sox5. The most significant hits can be obtained with the PFM Pax4.

Using the same dataset and prediction method as mentioned above we calculated the number of hits (in percent of the maximum possible hits) below a certain p-value cutoff (Figure 3). The percentage is decreasing linearly with the cutoff until a p-value of 10^−7^. Then the curve flattens presumably due to the low number of different PMFs effective for those p-value cutoffs. We also have been interested in how many different corresponding promoters are affected. Until a cutoff of 10^−6 ^all promoters receive a hit. When increasing the significance in a TFBS search from the default cutoff 10^−4 ^to 10^−7 ^(i.e. by the factor 1000), only half of the matrices will report hits from only 50 percent of the promoters. It is thus important to know down to which p-value cutoff a certain PFM is able to obtain hits. A table for the JASPAR CORE Vertebrata PFMs is given in Additional file 1.

### Comparison with similar tools

We have compared the capabilities of *D-Light* with eight similar publicly accessible services. While some of the tools use a pure HTML/JavaScript approach, others including *D-Light* implement a Java-Client software. We investigated regarding six properties: (i) The tool uses precalculated TFBS. (ii) It enables combinatorial queries, either by searching for co-occurrences of TFBS or clusters of TFBS or comparison between occurrence in a set of homologous sequences with the final goal to decrease false positive predictions. (iii) The tool provides access to sequences of multiple genomes. This is implicitly the case when multiple sequences can be uploaded. (iv) The tool accepts sequences uploaded by the user. (v) The tool accepts PFMs uploaded by the user. And (vi), the results can be downloaded in a textual, tabular form, or textual results can easily be copy-pasted from the corresponding web page for further processing by the user. The results are summarized in Table 1.

**Table 1:** Comparison with similar tools

**Tool**	**Foot**-	**TFBS**	**Precalc.**	**Sequence**	**PFM**	**Textual**	**Ref.**
	**print**	**comb.**	**sites**	**upload**	**upload**	**results**	
MotifViz	-	-	-	+	+	+	[26]
TargetExplorer	-	+	-	+	+	+	[27]
TFM-Explorer	-	+	-	+	-	+	[13]
CONREAL	+	-	-	+	-	+	[17]
MAPPER2	+	-	+	+	+	+	[18]
rVista	+	+	-	+	+	-	[28]
EELWeb	+	+	+	-	-	+	[11]
Toucan 3	+	+	-	+	+	+	[29]
*D-Light*	+	+	+	+	+	+	

A short survey of how the tools characterize themselves on their respective web page, the corresponding web links and some remarks are given in Additional file 2. Note that there are other similar software tools such as SeqVISTA [30] or GeneACT [15], which were not accessible or fully working at the time of writing the manuscript. We therefore could not properly evaluate these tools for adding them to the table.

### Application to a microarray dataset

Cheung et al. demonstrated the applicability of their software GeneACT [15] by using the microarray data set (GSE1692) deposited in NCBI Gene Expression Omnibus [31] by Cam et. al [32]. The GSE1692 set contains expression data of cell cycle dependent genes in T98G fibrosarcoma cells. Cheung et al. defined differentially expressed genes (positive set) and non differentially expressed genes (negative set) by f-test p-value cutoff of *p* < 0.05 and *p* > 0.7 respectively and provided the corresponding lists of genes as Additional file 1 to their publication. We extracted the list of genes, respectively the corresponding NM numbers and prepared the positive and the negative list by adding a ‘$’ sign to end of each NM number (NM_xxxxxxx\.) to enforce full length accession code matches in *D-Light* while ignoring the sequence version number.

Cheung et al. found that the E2F family binding sites are over-represented in the positive set. The JASPAR entry E2F1 represents the binding pattern of the E2F family members such as E2F-1 or E2F-4. The authors proved the correctness of the predictions for some of the genes of the positive set by conducting ChIP assays [15]. We wanted to know if one can get comparable results using *D-Light*. For this purpose we pasted the positive list to *D-Light* to query the TF annotations in hg19 (p-value cutoff 10^−4^) and downloaded the resulting data in a comma separated values (csv) file format and did the same for the negative list. From the GSE1692 positive set 767 genes were represented in D-Light, and 723 genes from the negativ set.

We then counted the occurrences of all annotated TFs, calculated the average occurrence per gene $\bar{P_{t}}$ and $\bar{N_{t}}$, where *t* is one of the TFs provided by *D-Light*. For each *t* we calculated the ratio $r=\bar{P_{t}}/\bar{N_{t}}$. The top ranking over-represented TF is indeed E2F1 (*r* = 1.41) followed by NFYA (*r* = 1.34) and USF1 (*r* = 1.26) which is in accordance to the results of Cheung and coworkers. The ratios delivered by our computational experiment, however, are rather low. We repeated this analysis for various p-value cutoffs. The results are shown on Table 2. At high p-value cutoffs no overrepresentation is observed. The lower the cutoff, the higher are the ratios. Unfortunately, the minimum reachable p-value of a certain PFM is limited and the predicted hits disappear below a certain p-value cutoff. In order to obtain evidence for the reliability of the ratios we applied the bootstrap function of R to calculate the 95% confidence intervals of the $\bar{P_{t}}$ and $\bar{N_{t}}$ values. In any case the average values are within the 95% CI.

**Table 2:** Overrepresentation of predicted TFBS in the GSE1692 data set

**p-value**	**E2F1**	**NFYA**	**USF1**	**MYC::MAX**
1∗10^−3^	1.08	1.02	0.98	0.99
5∗10^−4^	1.10	1.08	1.07	0.99
1∗10^−4^	1.41	1.34	1.26	1.15
5∗10^−5^	1.39	1.45	n/a	1.37
1∗10^−5^	n/a	n/a	n/a	1.71

Ratios of predicted TBFSs per gene are shown for different p-value cutoffs and four different top ranking TFs. n/a indicates, that the cutoff is below the minimal reachable p-value for the respective PFM.

We then performed queries with the same lists of genes, but now using the *D-Light* feature to cross-check if a certain binding site is also predicted in the homologous mouse gene (mouse genome version mm10). By doing so, the number of usable genes (i.e. genes where a homologous gene is defined by HomoloGene) reduces to 619 and 548 respectively. The ratio of over-representation increase to 2.45 for E2F1, which demonstrates the value of incorporating orthologous information to improve the quality of the predictions.

The TF occurrences counting in the csv formatted data files was performed using a simple Python script (available at http://biwww.che.sbg.ac.at/dlight/tools/overrep.py) but using Excel should be also feasible for an experienced Excel user. We extended the Python script to search for over-represented pairs of the TFBS with a minimum sequence separation of 10 and a maximum sequence separation of 100 base pairs. The five top ranking pairs are E2F1-NFYA, Mycn-USF1, MYC::MAX-USF1, USF1-USF1 and FOXC1-RREB1. Equipped with these results one now could use *D-Light* to search for other genes which have co-occurrences of e.g. E2F1-NFYA or query for other partnering factors for E2F1.

## Conclusions

*D-Light* is a platform independent client-server software to integrate, store and query annotation data for promoters for an arbitrary number of genomes. A major benefit is the smooth integration of user supplied small scale data with pre-assembled large scale data. *D-Light* complements other computational tools in the context of predicting and analyzing gene regulation.

Software components responsible for data import are written in Python and thus can easily be adapted to handle other annotation data than TFBS or other prediction methods thereof. Both, client and server are open source. The software can be installed locally in sensitive environments.

The analysis of a non redundant human promoter data set has shown that on average up and downstream TSS regions are equally covered with potential TFBS’s and that on average a ±2 kB region the most densely annotated one. However, *D-Light* is not restricted in this manner and may be set up with any promoter sizes.

For the next version of *D-Light* we consider to include the complete genome sequences which then should overcome the currently narrow definition of a promoter region. As shown in the array data use case example, some external scripting is required to search for over-represented TFBS in certain sets of genes. We will investigate also other potential use-cases and include the required procedures and statistical analyses directly into *D-Light*. Then, import and export of annotations and sequences in standard file formats such as gff3 will be an issue. Finally, links to other useful databases such as NCBI Nucleotide, NCBI Protein, PubMed or PDB will be established.

## Availability and requirements

**Project name:** D-Light

**Project home page: **http://biwww.che.sbg.ac.at/dlight

**Operating system(s):** Client platform independent, server requires Linux

**Programming languages:** Java, Python, C++

**Other requirements:** The client requires Java 1.6 or higher, for server see installation manual

**Any restrictions to use by non-academics:** none

Binaries and source is also provided in Additional files 3 and 4.

## Competing interests

The authors declare that they have no competing interests.

## Authors’ contributions

JL, TE, MF, SG, CL designed and implemented *D-Light*. CJZ performed the statistical analyses and the example applications. PL conceived the study, contributed to implementation and data analysis and wrote the text. All authors read and approved the manuscript.

## Supplementary Material

Additional file 1 **P-values for JASPAR PFMs.** Table with the lowest reachable p-value for the 130 JASPAR CORE Vertebrata PFMs. Click here for file

Additional file 2 **Comparison with similar tools.** Short survey of how the tools characterize themselves on their respective web page and the corresponding web links. Click here for file

Additional file 3 **Installation package.** Server and client software for local installation. Click here for file

Additional file 4 **Source code package.** Source code of server and client. Click here for file
