# Supplementary material for: D-Light on promoters: a client-server system for the analysis and visualization of cis-regulatory elements
Source: BMC Bioinformatics. 2013 Apr 24;14:140. doi: 10.1186/1471-2105-14-140 (PMC3685601; doi:10.1186/1471-2105-14-140)
Supplement: Additional file 2 — Comparison with similar tools. Short survey of how the tools characterize themselves on their respective web page and the corresponding web links. [file 1471-2105-14-140-S2.pdf]

TFM-Explorer

URL:

`http://bioinfo.lifl.fr/TFM/TFME/`

Description from the web page:

TFM-Explorer (Transcription Factor Matrix Explorer) is a program for analysing regulatory regions of eukaryotic genomes. It takes a set of coregulated gene sequences, and searches for locally overrepresented transcription factor binding sites.

rVista

URL:

`http://genome.lbl.gov/vista/rvista/submit.shtml`

Description from the web page:

Locates regulatory sequences in the user supplied data using comparative sequence analysis and transcription factor binding site search.

Remarks:

The rVista software is included in the "Vista Tools for Comparative Genomics" at `http://genome.lbl.gov/vista/index.shtml`.

A similar tool is rVista 2.0 at `http://rvista.dcode.org/` . In the operating mode comparable to rVista, only two sequences can be supplied.

CONREAL

URL:

`http://conreal.niob.knaw.nl/`

Description from the web page:

Identifies and visualizes conserved transcription factor bindings sites in orthologous promoters.

Remarks:

The upload is limited to two sequences. When a results page is saved as text, extracting the table requires some effort.

MAPPER2

URL:

`http://genome.ufl.edu/mapper/`

Description from the web page:

MAPPER is a platform for the computational identification of transcription factor binding sites (TFBSs) in multiple genomes, that combines TRANSFAC and JASPAR data with the search power of profile hidden Markov models (HMMs).

Remarks:

Instead of user PFM the TFBS characteristics can be uploaded as multiple sequence alignment from which MAPPER2 creates an HMM.

EELweb

URL:

`http://tkt_bsap.staff.cs.helsinki.fi/EELweb/`

Description from the web page:

Enhancer Element Locator, or EEL, is a tool for locating distal gene enhancer elements in mammalian genomes by comparative genomics.

Target Explorer

URL:

`http://luna.bioc.columbia.edu/Target_Explorer/`

Description from the web page:

Target Explorer automates the entire process from the creation of a customized library of binding sites for known transcription factors through the prediction and annotation of putative target genes that are potentially regulated by these factors.

Remarks:

The service is restricted to Drosphila, although any sequence and PFM can be uploaded. A PFM upload requires registration.

MotifViz

URL:

`http://biowulf.bu.edu/MotifViz/`

Description from the web page:

Motif Search and Visualization.

Remarks:

Allows to choose between four different motif scanners. Although the upload of multiple sequences is allowed, the sequences are treated as unrelated independent instances.

TOUCAN 2

URL:

http://homes.esat.kuleuven.be/~saerts/software/toucan.php

Description from the web page:

TOUCAN is a workbench for regulatory sequence analysis on metazoan genomes : comparative genomics, detection of significant transcription factor binding sites, and detection of cis-regulatory modules (combinations of binding sites) in sets of coexpressed/coregulated genes.

Remarks:

Comprehensive tool which integrates many different primary tools from the web.
