# Supplementary material for: D-Light on promoters: a client-server system for the analysis and visualization of cis-regulatory elements
Source: BMC Bioinformatics. 2013 Apr 24;14:140. doi: 10.1186/1471-2105-14-140 (PMC3685601; doi:10.1186/1471-2105-14-140)
Supplement: Additional file 3 — Installation package. Server and client software for local installation. [file 1471-2105-14-140-S3.zip › dloprom-1.1/manual.pdf]

D-Light

on Promoters

User Documentation

Version: 1.1
Date: February 15, 2013
Contact: d-light@fh-hagenberg.at

The following people contributed to the project (alphabetical order):

Sophie A. Blank
Tobias Ehrenberger
Monika Freudenberger
Simone Gschwandtner
Peter Lackner
Josef Laimer
Carina Lebherz
Gerald Lirk
Paul Neulinger
Alexander P. Seitinger
Alexandra M. Simader
Clemens J. Zuzan

Contents

1 Introduction **4**

2 Background **5**

2.1 Short introduction to TFs 5

2.2 Data sets 7

2.3 Annotation Method 7

3 Using the *D-Light* Client GUI **8**

3.1 Before you start 8

3.2 Terms and Naming Rules 8

3.3 Main GUI Elements 9

3.4 Starting the GUI and logging in 9

3.5 Query the database 10

3.5.1 New Query 11

3.5.2 Stored Queries 13

3.5.3 Datasets 14

3.6 Viewing Results 14

3.7 Annotation Viewer 14

3.7.1 Selection History Panel 18

3.8 Upload/Delete User Data 18

4 Server Installation **23**

4.1 System Requirements 23

4.1.1 Hardware 23

4.1.2 OS Versions 23

4.1.3 Software 23

4.2 Server setup 24

4.2.1 Software Installation 24

4.2.2 Initial data setup 25

4.2.3 Prepare a *D-Light* website 26

1 Introduction

D-Light on Promoters is a client-server based system for quering and viewing annotation data on promoter sequences. By default, the underlying database is filled with transcription factor binding site (TFBS) data during server setup. Users may add also other arbitrary annotations at any time and query them in the context of the existing ones.

Why this name, *D-Light*? The original technical aim of this software was to implement a light-weighted sequence annotation viewer, which uses the DAS protocol for retrieving the annotation data from a server. Since then, our focus has changed and we aimed to develop a user-friendly, nicely cross linked and well performing system to handle annotation data for DNA sequences, in order to put some light on DNA.

Right after the server installation (or using our public *D-Light* server) a user can query precalculated data of predefined promoter and PFM (Position Frequency Matrix) sets and view them in a graphical manner. For more advanced usage, however, users may add own data sets (PFMs, promoter sequences, precalculated annotation sets). *D-Light* allows for querying single TFBSs or pairs of TFBSs. Cross-genome searches verify the matches in homologous genes.

For sensitive and/or multi user environments the system provides a user management such that users can create personal accounts and upload/remove personal data, which are invisible for other users but can be queried in the context of the public content.

Hence there are two different type of users: A “public” user, which is enabled by default, and personalized users.

What can the public user do?

- Query and view all genes or selected genes for all binding sites or selected binding sites fulfilling some score or p-value criterion.
- Query/view pairs of sites within a certain distance, with one PFM fixed or both PFMs fixed.
- Check if these match pairs also occur on an orthologous gene in a reference genome.
- Download results as csv or gff3 files.

What can a personalized user additionally do?

- Send a custom PFM to the server and letting the server find the TFBSs.
- Send a custom promoter to the server, letting the server find the TFBSs.
- Add custom annotation data which represent TBFSs for certain PFMs calculated with any external method.
- Sending custom annotation data which do not represent TBFS data, such as data on methylation, histon binding, etc.
- Delete personal data.

If you want to be informed about bug fixes, new features or new versions of *D-Light*, drop us an E-mail at:

d-light@fh-hagenberg.at

Any suggestions, bug reports, feature requests, a.s.o. are also very welcome.

2 Background

2.1 Short introduction to TFs

Regulation of gene expression is mediated by proteins termed Transcription Factors (TFs), which bind to DNA similar to a key in a locked switch. The enhancer/promoter regions upstream of a coding gene constitutes the main zone of binding. The various TFs take influence on the likelihood of an RNA-Polymerase joining the promoter and initializing transcription. Combinations and aggregations of transcription factors fine-tune the time point of release and the amount of the transcribed RNA and thus of the gene-product.

Below we summarize the structural motifs found in the DNA-binding domains of TFs and present some examples, how the motifs are arranged in the DNA-binding domains and interact with DNA.

DNA binding is accomplished by a few structural motifs. An α-Helix usually hosts the aminoacids which make the specific contacts to the bases in the major grove. Unspecific binding is achieved by the basic amino acid sidechains of Lysine and Arginine interacting with the phosphate-deoxyribose backbone of the DNA. Thus in all motifs at least a part shows increased amount of Lys and Arg, often called basic region.

One of the first identified motifs is the helix-turn-helix (HTH) motif (Fig 1a) found in homeodomains (Fig 1b). The blue helix contains several basic residues. In Fig 1c the binding of the basic helix of the homeodomain from antennapedia in the major groove is shown. Homeodomains are found exclusively in eukaryotes and predominantly involved in differentiation.

A second DNA binding motif is the zinc-finger. Zinc fingers coordinate a zinc ion with Histidine and Cysteine residues (Fig 1d). The ion stabilizes the folded motif. The specific contacts are again contributed by the helical region. Zinc-finger usually appear in repeats in DNA-binding domains (Fig 1e). The number of repeats can differ considerably in different TFs. Also functionally inactive degenerated fingers can be found beyond the repeats. HTH and zinc fingers have also be found in combination in a single TF, as e.g. DNA binding domain of the glucocorticoid receptor, which binds as dimer (Fig 1f).

For the leucin zipper motifs (Fig 1g) dimerization is mandatory for DNA binding (Fig 1h). The monomer consist of a single, long α-Helix, again with an Arg/Lys-rich basic region (Fig 1g, colored in blue) and a leucin rich region (Fig 1g, colored in red), which is responsible for dimerization. Similar, the basis helix-loop-helix (bHLH) consist of a long helix containing the basis DNA binding region (Fig 1i). The upper part of this helix and the loop-helix conformation are responsible for dimerization. This region enwinds the corresponding region of the second monomer in form of a four helix bundle. Finally, a kind of fusion of the bHLH and the leucin zipper is the bHLH-zipper motif (Fig 1j) as found in Myc-Max and Mad-Max heterodimers.

These example show that there is a certain diversity of binding modes which reflects in the properties of the binding sites, such as length and symmetries. The specificity of the different TFs is thought to be reasonably reflected in the corresponding PFMs. This is also what we consider as true enough to use PFMs in our *D-Light* system.

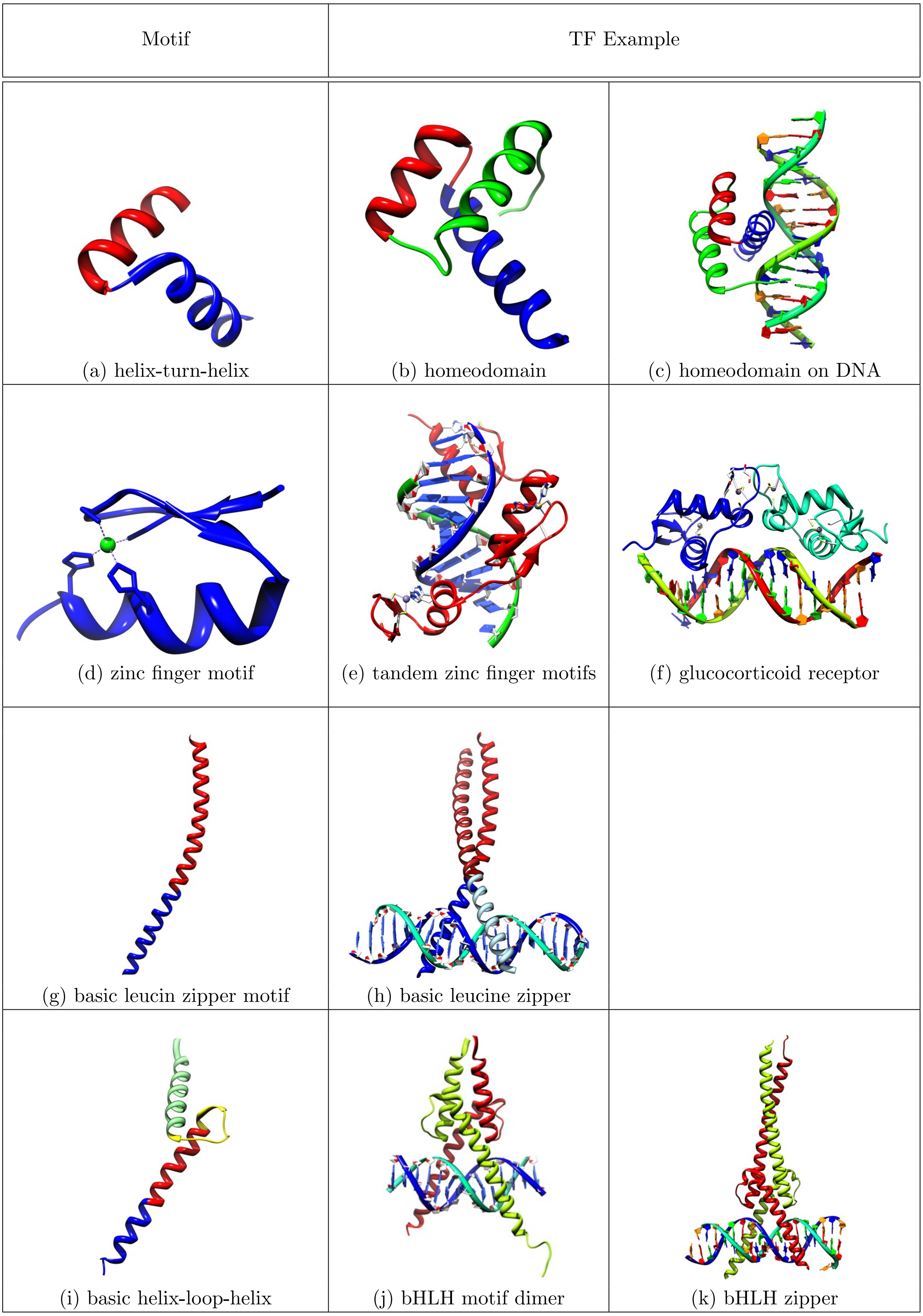

(a) helix-turn-helix
(b) homeodomain
(c) homeodomain on DNA
(d) zinc finger motif
(e) tandem zinc finger motifs
(f) glucocorticoid receptor
(g) basic leucin zipper motif
(h) basic leucine zipper
(i) basic helix-loop-helix
(j) bHLH motif dimer
(k) bHLH zipper

Figure 1: DNA binding motifs and their arrangement in TFs.

2.2 Data sets

The default PFM dataset is obtained from the JASPAR database
http://jaspar.genereg.net/html/DOWNLOAD/

The genomics sequences used to prepare the promoter regions are downloaded from the UCSC database.

The homology information is retreived from the NCBI ftp site
ftp://ftp.ncbi.nih.gov/pub/HomoloGene/current

2.3 Annotation Method

The built-in annotation method applies a simple scoring scheme. By default, the raw matrix data are converted into log-likelyhood ratio scores. Let $n_{a,i}$ be the raw counts in the PFM for base $a \in \{A, C, G, T\}$ at column position i. To each $n_{a,i}$ value a pseudocount (default 1) is added and the resulting values are normalized by the colunm sum resulting in relative frequencies $f_{a,i}$. We then calcuate a log score value

$$s_{a,i} = \ln \frac{f_{a,i}}{b_a}$$

using the background probabilities b_a (default $b_A = b_C = b_G = b_T = 0.25$).

Given a certain PFM of length N and log score values counts $s_{a,i}$ we first calculate the maximum reachable score.

$$S_{max} = \sum_{i=1}^{N} \max s_{a,i}$$

Then we obtain the current absolut score for the PFM given a certain DNA sequence $a_1, a_2, ..., a_N$:

$$S_{abs} = \sum_{i=1}^{N} s_{a_i}$$

The corresponding p-value $p(S_{abs})$ is calculated using the method of described in by [**?**].

Finally, the S_{abs} is converted to a relative score ranging from 0 to 100: $S = S_{abs}/S_{max} * 100$.

To obtain a manageable amount of data, only sites with a p-value ≤ 0.002 are stored in the database by default.

Note that the parameters pseudocounts, background distribution and p-value cutoff can be changed at server setup time.
Other scanning methods can be utilized on the server side via a Python wrapper script. An example wrapper for FIMO from the MEME suite is included in the server distribution.

3 Using the *D-Light* Client GUI

3.1 Before you start

The *D-Light* client is based on Java and is provided as Java applet for web browsers, as Java web start client and as standalone Java program. All versions require Java JDK or JRE 1.6 (or later). For using the browser applet version enable Java for your browser. We tested the *D-Light* applet with current versions of Firefox, Internet Explorer and Safari. Other browsers supporting Java should also work.

Pros and cons of the different versions:

	pros	cons
applet	does not require any setup<br>user always gets latest version	limited fixed size<br>entry fields may loose focus
web start (WS)	GUI is resizable<br>does not require any setup<br>user always gets latest version	requires WS support on the client<br>longer start-up time
stand alone	GUI is resizable<br>can be linked to desktop	requires a bit of setup<br>latest version has to be installed by hand

For all client versions a network connection to the server is required. If client and server are on different systems separated by a firewall be sure to open the TCP ports 8080 and 1099 for server communication. Note that the ports might be different on a customized installation (see section 4, "Server Installation"). For details on the stand alone client refer to the bundled README file.

3.2 Terms and Naming Rules

We follow roughly the DAS [**?**] nomenclature for *D-Light*. Sequences are annotated with features and this information is stored in *D-Light*.

A **feature** is a region on a particular sequence with certain properties. Examples for types of features are predicted transcription factor binding sites (TFBSs), experimentally determined TFBSs, histone binding sites etc.

Features are generated by a certain **annotation method**. By default, *D-Light* has a built-in annotation method called pyfscan (see section 2.3, which uses PFMs provided via the JASPAR [**?**] database and predicts matches of the PFMs on all supplied promoter sequences. Users may add features which are generated by other arbitrary methods. E.g. one could use FIMO [**?**] or ScanACE [**?**] to predict binding sites and upload these data. Or an experimentalist provides a set of known binding sites which is then considered as a certain annotation method.

Feature ACCs (ACC = accession code) have to be unique. E.g. the JASPAR naming standard is `MAnnnn.n`, where $n \in \{0..9\}$. `MA0031.1` is the JASPAR PFM ACC for FOXD1 and may not be used for anything else. The same holds true for sequences and sequence ACCs. By default we use NCBI RefSeq ACCs, e.g. `NM_003636.2`

Different annotation methods should use the same feature ACCs if they mean the same type of feature, e.g. binding sites for FOXD1 should always have the ACC `MA0031.1`, if they use the JASPAR PFM `MA0031.1`.

At the moment only JASPAR ACCs (MAnnnn.n) and NCBI RefSeq ACCs (NM_nnn...) have valid link-outs to the original databases in the *D-Light* GUI.

3.3 Main GUI Elements

Several windows are equipped with a refresh button: . Pressing forces the client to contact the server for asking if a pending operation provides any new data to be visualized in the current window.

The *D-Light* GUI allows to query and visualize pre-calculated annotation data as well as adding new data to the private data sets. The major GUI windows are:

- **Login** Login and/or add a new user.
- **Main Window** Enter a query or add new data. After selecting the mode the corresponding widget appears in the lower section of the main window.

GUI text fields are either mandatory (labels in ***blue italic font***), optional (**labels in normal font**) or disabled (**labels in grey color**). Disabled fields appear when logging in to the non-personalized public account. All mandatory fields are pre-filled with a default value. Please revise these fields carefully.

3.4 Starting the GUI and logging in

Start your favorite web browser and open the URL (As you are reading this manual you either got it from our public web server or from your local *D-Light* installation. Thus you know your *servername*):

`http://`*servername*`/dlight`

You should get this page:

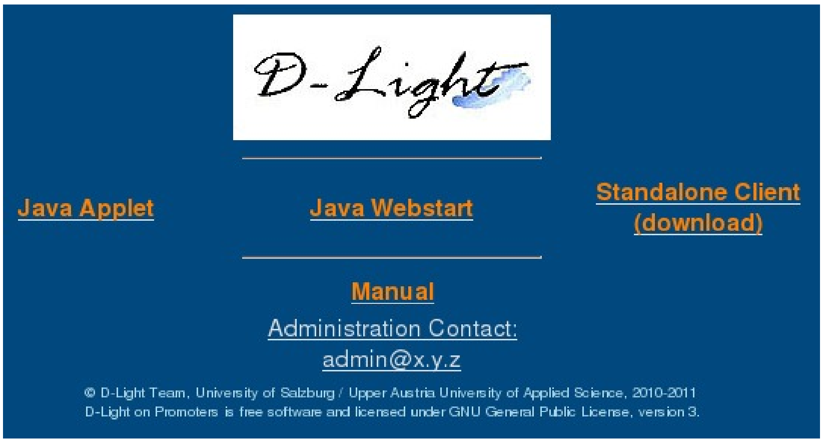

We recommend to use either the web-start version or the standalone version. If you decide to use the standalone version, download the corresponding client.zip file, unpack it, and check the README file for further instructions.

The usage of all client variants is the same. You first need to log in. *D-Light* provides the default user "Public", which has restricted access privileges. Click at "Public Access" in the **Login** widget for logging in as this user.

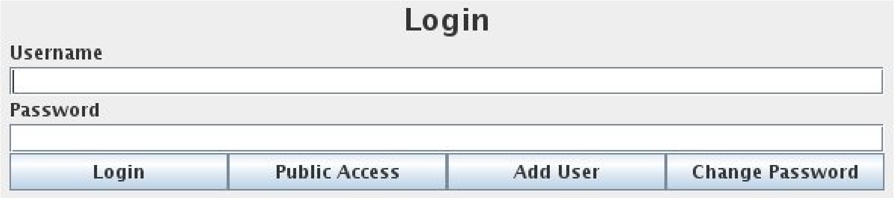

If not disabled by the *D-Light* administrator, you can create your personal account by pressing the "Add User" button. For the username only the characters a-z,A-Z,0-9 are allowed.

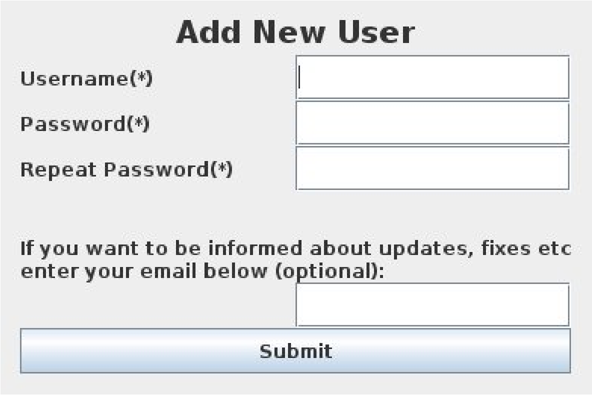

If you forgot your password please contact your *D-Light* administrator. The e-mail address for our public *D-Light*server is shown on the corresponding web page.

We enabled the **Public** user account for the purpose of testing and exploring the system. We use this account for teaching. The **Public** user is not allowed to upload data to ensure a consistent database. *On our web server queries from the Public user are deleted after one month!*

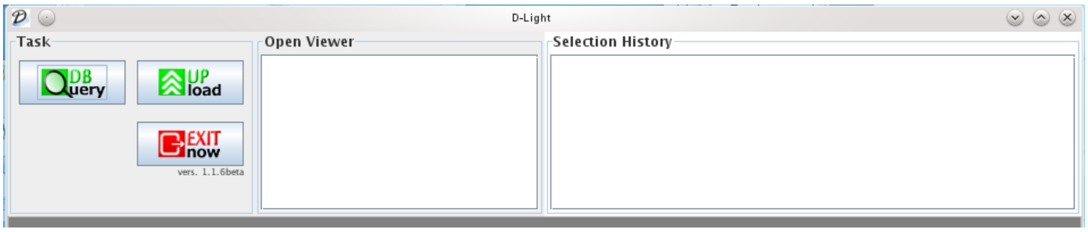

After logging in, the *D-Light* GUI appears with three panels in the upper part. With the *Task* panel you either enter the query mode, the data upload mode or leave the application. *Open Viewer* lists all available viewer instances. *Selection History* lists detailed information of selected features.

3.5 Query the database

The widget is organized in three tabs, *New query*, *Stored Queries* and *Datasets*.

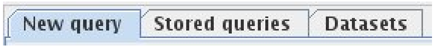

New query allows to specify a new query from scratch, which then either can be (i) just stored for future use or (ii) immediately executed and visualized. Each query is stored on the server and can be accessed for re-running or modification using the *Stored Queries* tab. The *Datasets* tab can be used to retrieve information from the database which is not directly related to annotated TFBS such as promoter entries or server settings.

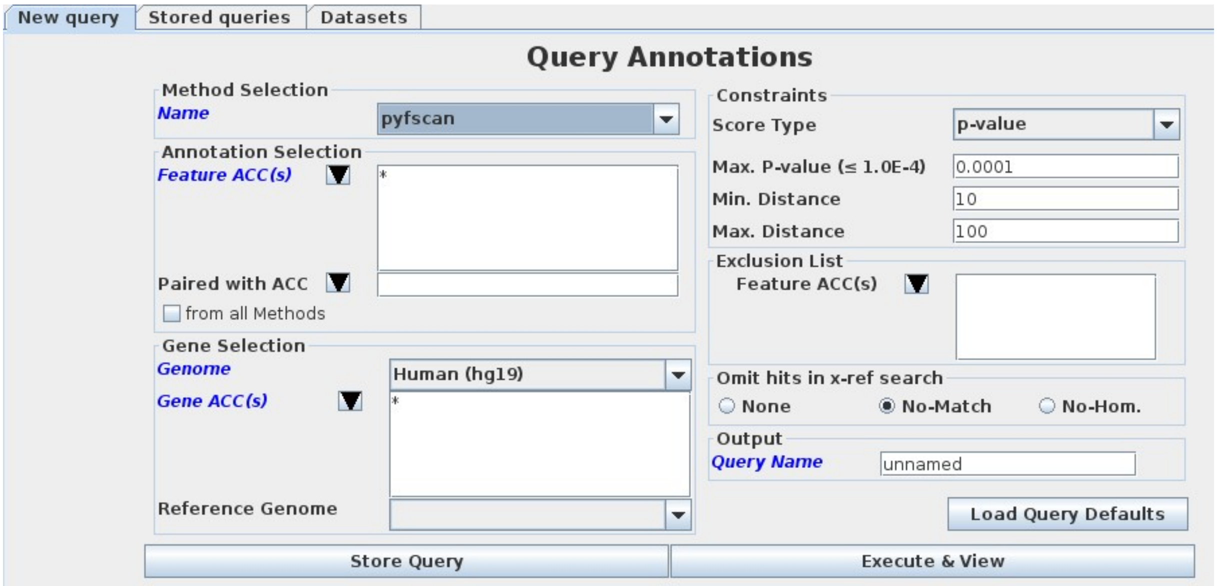

3.5.1 New Query

The logic is as follows:

1. Select an annotation method.
2. Select feature ACCs (or combinations thereof)
3. Select genome(s) and genes.
4. Select constraints
5. Select feature ACCs to be excluded from search
6. Name your query
7. Store/execute the query

Step 1, Select an annotation method

The annotation method determines the available scores and matrices. The built in method (pyfscan) provides a log odd score, the normalized score thereof as well as a p-value of the log odd score (See section 2.3) for details).

Step 2, Select Features

Enter one or more ACC(s). By default, an asterisk '*' is written there, which means "select everything".

The field ***Matrix ACC(s)*** enables a regular expression like syntax. E.g. MA003 matches all PFM IDs which start with MA003, i.e. MA0030 to MA0039. With 'or' operations it is possible to select several matrices: `MA0030|MA0050` applies the query for both given PFMs. Alternatively, a list of matrices can supplied. Giving `MA0030` and `MA0050` in separate lines is equivalent to `MA0030|MA0050`. Therefore one may simply copy–paste lists of accession numbers from a text editor window to the ***ACC(s)*** field. For selecting all PFMs use the asterisk '*'. The same holds true for ***Gene ACC(s)***.

Alternatively, by clicking on the down arrow ▼ button left to ***Feature ACC(s)***, the list of available matrices appears in a pop-up window. Such selection lists appear throughout *D-Light* and are usually equipped with a *Filter* function. Strings you type there are searched in all columns in the table. Only rows which match the filter string remain in the list.

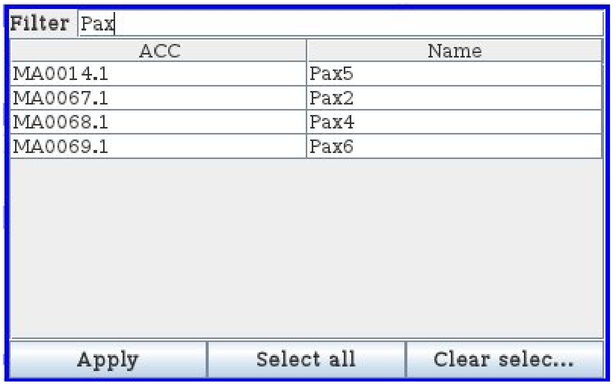

The filter string in filtered lists is case insensitive. Moving the mouse over the "Filter" line shows the total number and the currently affected number of list entries. Depending on the context, where such a list appears, you may finally select a single line (Left-Mouse-Button), a continuous block of lines (Shift-Key + Left-Mouse-Button) or several lines (Control-Key + Left-Mouse-Button).

Then you may enter the score (resp. p-value/e-value) cutoff. We assume that highers scores are better and lower p/e-values are better. The default cutoff are set to zero for scores and one for p/e-values (means no restriction).

Note that the default p-value cutoff for the built-in method is 0.0001. Hits with a higher p-value are not stored in the database. Therefore some PFMs may show no hits at all. To determine those cases use the "Download Dataset" feature and download the *Annotations* (see page 14). Look in the resulting file for lines containing 'hits=0'. The p-value cutoff can only be changed during server setup.

Features predicted by an external method may have larger p-values. When you import such annotations, **no cutoff** is applied. Thus all uploaded annotations are included.

Use the field **Paired with ACC** for querying pairs of PFMs which appear in close proximity on a promoter. **Paired with ACC** can only be a single matrix ACC. For pair queries the constraints **Min. Distance** and **Max. Distance** are applied. The numbers are the space between the end of the 3' binding site and the start of the 5' binding site. The **Paired with ACC** can be either at the 3' or 5' side of the other feature. There are two modes of operation for the **Paired with ACC** option. Normally, the available ACCs are the ones accessible in the selected annotation method. By clicking the check-box "(from all Methods)" all feature ACCs known in *D-Light* are provided for selection and *no* score cutoff is applied to the **Paired with ACC**. This e.g. allows to search for TFBS located near a user defined feature. Negative values for **Min. Distance** represent the number of bases allowed to overlap, e.g. "-2" means that the begin of the first site may overlap by two bases with the end of the second site.

Step 3, Select genome(s) and gene(s)

As mentioned before, *Gene ACC(s)* can be a single one, a set of ACC(s) or all genes from a certain genome (*). The genome can be selected in the ***Genome*** drop down menu.

D-Light supports cross genome queries. An internal cross reference table derived from HomoloGene [**?**] links the orthologous genes between the different genomes. Once a *Reference Genome* is selected, a match in the main genome is cross checked in the orthologous gene in the reference genome.

The output of the cross reference genomes search can be customized with *Omit hits in x-ref search*. *nohom* means, that genes with no known homologs are omitted. *nomatch* means, that in addition hits with no corresponding match in the homologous promoter are omitted.

Step 4, Select constraints

Depending on the annotation method score values and/or p/e-values are available. Scores are assumed to be increasing for better matches, p/e-values are assumed to be decreasing for better matches. Once the **Score Type** is selected, the cutoff field shows up as **Max Score** or **Min p/e-Value**. The fields **Min Distance** and **Max Distance** denote a distance cutoff along the DNA sequences for pair searches.

Step 5, Select features to be excluded from search

Optionally, a list of PFM ACC(s) can be supplied, which will be excluded from the search. This is useful e.g. for frequently bindings PFMs.

Step 6, Name your query

Queries are enumerated by the server. However, it is recommended to add a meaningful query name for easy identification of your queries.

Step 7, Store/Execute your query

The *Store Query* button just saves your query parameters on the server under the current login. *Execute and View* in addition executes the query and launches the annotation viewer.

3.5.2 Stored Queries

This tab shows all queries entered by the current user in a filtered list. You can select one or two queries at a time. Clicking on "Execute and View" executes the selected queries and submits the results to the viewer. The selection of two queries enables a comparative analysis in the viewer. This is useful for cross-genome searches, comparison of two different promoters or the comparison of runs with different parameters.

The meaning of the other buttons is:

Delete Selected Queries:	Delete the currently selected queries.
Delete All Queries:	Delete all stored queries.
Clear selection:	Unselects everything.
Edit:	Shows the "Scan Annotation" tab and fills the corresponding fields with the value of the (first) selected query.

3.5.3 Datasets

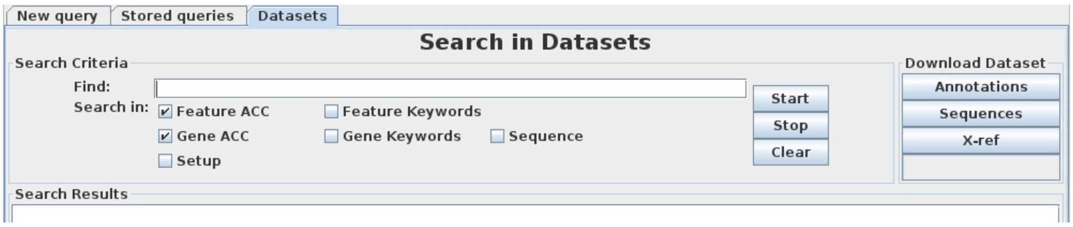

The “Datasets” tab provides keyword or accession number searches in the different data sets stored in *D-Light*’s MySQL database and download options of data from the database.

Queries can be performed in (1) PFM (feature) ACCs, names or descriptions, (2) sequence ACCs, names or descriptions or (3) the sequences themselfes. Matching records are shown with their full content (e.g. PFM data, or promoter sequences). In case the checkbox *Sequence* is selected, a simple substring search is performed in all stored DNA sequences. Note that only the stored sequence direction is searched, not the corresponding complement. Note that similar queries can also be performed via the “Inventory” tab and the filter function but for the private data only.

Searches in the ’Setup’ show the configuration of the default data-set during server installation. To view the complete set of setup parameters enter ’`.*`’ in the search field.

There are three buttons in the “Download Dataset” pan. With “Annotations” you get all features (PFMs, etc.) currently stored in *D-Light* (public and private ones). “Sequences” delivers a multi-fasta file containing all sequences for a selected genome (public and private). Finally “X-ref” provides a tab-separated list of gene-ACCs which are defined as being homologous in different species, one homologous family per line. The x-ref file is especially useful if a user intents to upload new sequences e.g. to the human genome and want to supply homology information to regarding the other *D-Light* organisms. Then this list can be extended with the corresponding information for the new sequences and submitted to *D-Light* via “Upload X-ref”.

3.6 Viewing Results

Every time a query is executed a new viewer instance is created. The instances are listed in the ’Open Viewer’ list, labeled with ID and name of the query. To switch the viewer instance click on the corresponding line.

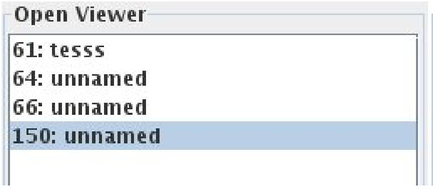

3.7 Annotation Viewer

The *D-Light* annotation viewer displays query results. On the left side there is a zoom- and scroll-able hit viewer panel ① . Each hit is represented by a small rectangle. On the right side ⑧ there is a tab panel for either selecting a certain gene, changing display styles or data export. For displaying the annotation data we assume that all supplied sequences are denoted in direction of transcription. The TSS is marked by a green triangle. The coding sequence is then at the right side of the TSS. The start of the coding sequence is marked with a yellow triangle. For several genes, the TSS in not known. To still

include such genes, the CSS is used instead as “zero” point. In that case the triangle is shown in red. In the figure below, the a minus strand gene is show, indicated by **(r)** right after the accession code. A gene on the forward strand is indicated by **(r)**. With the context menu one can switch between viewing and numbering in direction of transcription or genomic direction and numbering (see below). A reversed strand gene view then is rotated by 180°.

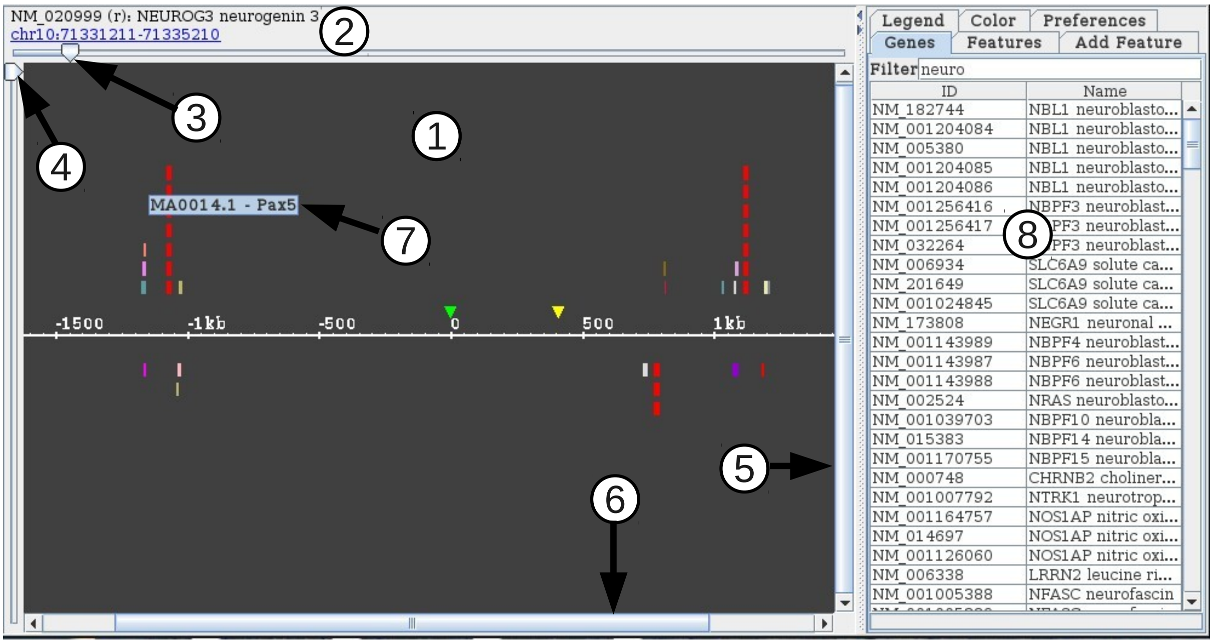

The currently displayed gene is described in the header ② . Accession codes (blue) are linked to the NCBI database. If a chromosomal location was supplied with the promoter sequences, a link to the UCSC genome browser appears in blue. There are two sliders for zooming in/out horizontally ③ and vertically ④ . By default, everything is within a visible area. When zooming in, the scroll-sliders ⑤ and ⑥ become active. If the mouse pointer is located over a certain annotation rectangle a tool-tip pop-up ⑦ shows ACC and name of the corresponding feature. You can select a certain hit by right mousebutton clicking on it. Detailed information about the hit is then shown in the *Selection History* panel.

In pair searches all pairs of hits are displayed. This will generally lead to stacks of rectangles, at least for the fixed “Paired with ACC”. Therefore, the size of the stack indicates in how many pairs the feature is involved.

A right mouse click in the viewing area opens a context menu:

Show coordinates relative to TSS	:	display in transcriptional direction
Show genomic coordinates	:	display in genomic direction
Show/hide features from method	:	display/hide groups of features
Copy sequence	:	copy sequence to clipboard
Copy annotation list (csv)	:	copy annotations to clipboard
Copy Image	:	copy current view to clipboard
Save annotation list (csv)	:	save annotations as csv file
Save annotation list (gff3)	:	save annotations as gff3 file
Save list + sequences (gff3)	:	include also the sequences
Save image	:	save current view in image file
Print (PostScript)	:	send view to printer

In case of a cross-species search using a reference genome an additional menu item “Filter homologs in lower panel” (“Filter homologs in upper panel”) appears in the context menu. This sets the gene filter in the respective other viewer to the homologous ones. This can be reset by removing the filter string.

The “Show/hide features from method” menu displays a submenu with annotation methods which allows to show or hide features provided by a certain method. The submenu is organized in two parts, separated by a horizontal line. The upper part refers to exactly the features which result from the query produced the current view. The lower part refer to features from methods which are show by default, e.g. the “seqfeat” TSS and CSS.

It could look like this, if your query was based on pyfscan and you have uploaded ENCODE data which are located in the current promoter:

☐ pyfscan

☐ ENCODE
☐ seqfeat

Both, “Copy List” and “Save List” generate a list of the current annotations of the displayed gene. Two formats are supported, csv (values separated by tabs) and ggf3. If the display is set to ’transcriptional direction’, the feature positions are relative to the 5’ end of the displayed sequence. If the display is set to ’genomic direction’, genomic coordinates are utilized. Saving in **gff3** format **always** generates **genomic coordinates**.

The panel ⑧ on the right side contains a number of tabs to browse through the query hits, add own features, view a legend and change display styles. The gene ACCs and the corresponding description is listed in the **Genes** tab. The **Filter** works as described above. E.g. the string “Neuroblast” would display all genes having the substring “neuroblast”” in their name:

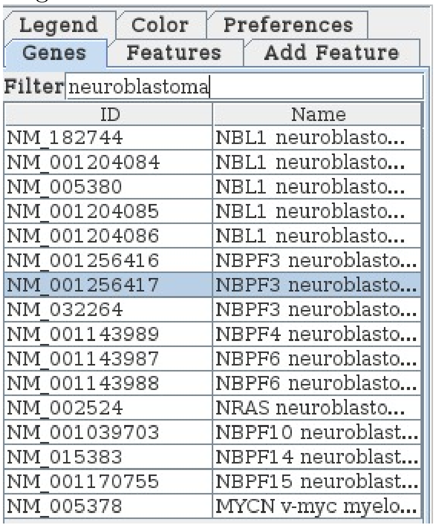

ID	Name
NM_182744	NBL1 neuroblasto...
NM_001204084	NBL1 neuroblasto...
NM_005380	NBL1 neuroblasto...
NM_001204085	NBL1 neuroblasto...
NM_001204086	NBL1 neuroblasto...
NM_001256416	NBPF3 neuroblasto...
NM_001256417	NBPF3 neuroblasto...
NM_032264	NBPF3 neuroblasto...
NM_001143989	NBPF4 neuroblasto...
NM_001143987	NBPF6 neuroblasto...
NM_001143988	NBPF6 neuroblasto...
NM_002524	NRAS neuroblasto...
NM_001039703	NBPF10 neuroblast...
NM_015383	NBPF14 neuroblast...
NM_001170755	NBPF15 neuroblast...
NM_005378	MYCN v-myc myelo...

The list of genes can be sorted by clicking on the column headers. The **Genes** tab owns the following context menu:

Copy gene list	:	copy tab separated list to clipboard
Save gene list	:	save list of genes to file
Save annotation list (csv)	:	save annotations as csv file
Save annotation list (gff3)	:	save annotations as gff3 file
Save list + sequences (gff3)	:	include also the sequences

The **Features** tab shows all distinct features annotated in the displayed gene. A left mouse button double-click on a feature displays all occurrences for that feature. A double-click on an occurrence selects that occurrence in the graphics and centers the displayed gene accordingly.

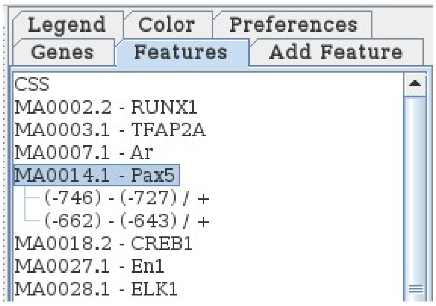

The **Feature** tab owns the following context menu:

Copy list	:	copy tab separated feature list to clipboard
Copy list incl. positions	:	the same including positions
Save list	:	save list to csv file
Save list incl. positions	:	the same including positions

The **Preferences** tab allows for setting the viewers background color, the pointers precision area (number of pixels around the cursor tip grepping for selection), and the selection style (default is a red "Outlined" hit rectangle, "Highlighted" changes the selected hit to red color and finally "None" means no outline/color change at all. The "Zoom from" defines the anchor position on zooming in.

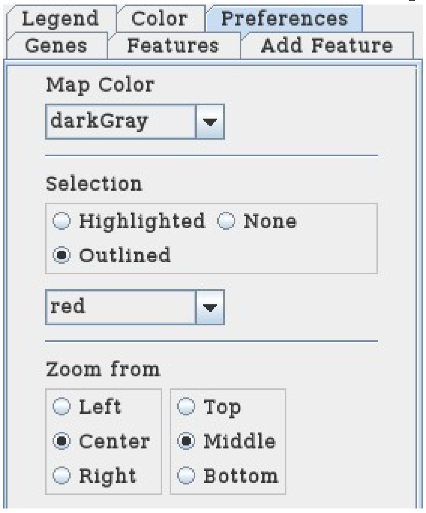

A number of different coloring options are available through the **Color** tab. **Color By None** means uniform coloring of the hits. The color can be changed by **Change Annotation Color**.

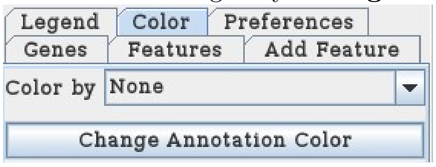

Color By Score colors by associated feature score values.

Color By Feature means that each matrix gets a different color. There are only 22 different colors available. Different features thus may get the same color. Use the **Legend** tab to view the color codes. If the coloring is "by feature", the **Legend** tab owns the following context menu:

Copy list	:	copy tab separated list to clipboard
Copy list incl. RGB values	:	same including RGB color values
Copy image		
Save list	:	save list to csv file
Save list incl. RGB values	:	same including RGB color values
Save image		
Print (PostScript)	:	send view to printer

3.7.1 Selection History Panel

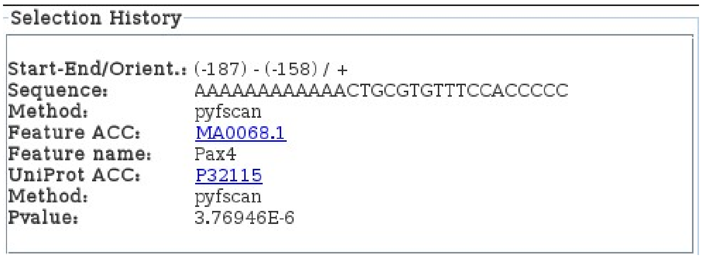

Each click on a feature in the viewer window displays a detailed feature info in the *Selection History* panel. Start/End are given relative to the assumed TSS (on the right side). If a pair search has been performed, the pairing feature ACC is given in the row "Pairs with". Underlined blue words are link-outs to the JASPAR database and the UniProt database respectively.

3.8 Upload/Delete User Data

The "Upload" button opens a multi-tab widget which enables the upload of PFMs, promoter sequences, binding site data or other annotated features.

The "Inventory" tab

is used to list all user supplied data. Sub-tab "Annotations" displays the type of annotation supplied by the user. We distinguish between PFM related annotations and general annotations. The former are derived by using a certain scanning method with PFMs to predict their potential binding sites. The sites have an associated score and/or p/e-value. The purpose of general annotations is to add information such as experimentally determined binding sites, methylation, etc, which subsequently can be displayed together with query results. Sub-tab "Sequences" lists promoter sequences. Sub-Tab "Features" lists the location (sites) of PFM related or general annotations on the promoter sequences.

Selected entries can be deleted. This removes all data on the server related to the selected entity. When deleting a certain PFM, also the corresponding binding sites are removed.

Note that only personal data can be removed by a user.

The "New Matrix" tab

allows the upload of a new matrix. Once the matrix is on the server, the server automatically starts a binding site scan on all public and personal sequences and adds them to the database. This may take a few minutes. You may continue to use *D-Light* and you will be informed once the server is ready with the new annotations.

You need to fill in the matrix data, a matrix ID and a matrix name. The matrix data have to be given in JASPAR style format, e.g.:

```
A  [ 3  0  0  0  0  0 ]
C  [ 8  0 23  0  0  0 ]
G  [ 2 23  0 23  0 24 ]
T  [11  1  1  1 24  0 ]
```

For a ***Matrix ID*** please don't use spaces! We recommend JASPAR style IDs, e.g. *MM0001*. The matrix ***Name*** is used to identify your matrix by keywords, e.g.: *Refined PAX5 matrix*).

The remaining optional fields resemble information commonly used in the JASPAR database.

The "New Sequence" tab

allows to add a single new DNA sequence to one of the currently stored genomes.

Supply an accession number ***ACC*** (don't use spaces! We recommend NCBI style ACCs, e.g. *MS_0001*. Also describe your sequence in the ***Name*** field. Optionally specify the corresponding genomic position, i.e. chromosome, location, strand, transcription start (TSS), start of the coding sequence (CSS) and the UniProt ID of the encoded protein. If given (and within the given chromosomal location range), TSS and CSS are marked in the viewer and TSS becomes the axis origin.

The sequence must be supplied in one letter code. Codes other than A,C,T,G or N are converted to N. A fasta style header line may be given but is not used by *D-Light*.

The optional field "Orthologous Genes" allows to supply a list of gene ACCs which are considered to be homologous to supplied promoter's gene in the other genomes. For getting the correct gene ACC we recommend to use "Query Dataset" tab. The supplied list is only checked for availability, not for usefulness. The list of orthologous genes enables user supplied promoters to be used in cross genome searches.

```
> ID and name are taken from the GUI
ggcctaatcgg.....
```

Once the sequence is on the server, the server automatically starts a binding site scan with all public and personal PFMs and adds them to the database. This may take a few minutes. You may continue to use *D-Light* and you will be informed once the server is ready with the new annotations.

For displaying the annotation data we assume that all supplied sequences are denoted in direction of transcription, i.e. that the coding sequence is *always* on the right side. Please consider this for your sequence and supply the reversed complement for minus-strand genes.

The "New Annotation Method" tab

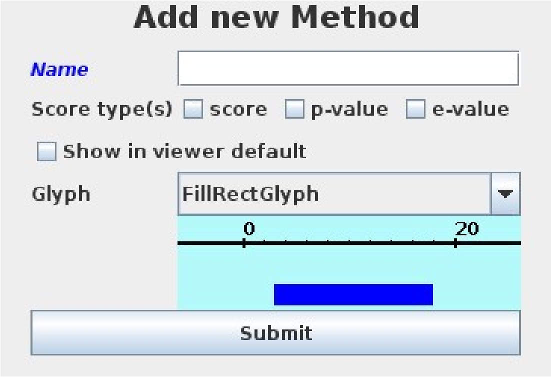

allows to specify a new external method which delivers annotation data (features). This can be either a prediction method or experimental data.

First, supply a name for the new type of data or annotation method respectively (e.g. FIMO, ScanACE, Methylation, Known-Site). Then specify which types of scores are provided by the new method, if there are any. *D-Light* considers these setting when you upload new features.

My selecting "Show in viewer by default" the uploaded features are displayed in the viewer widget whenever it opened.

Finally, select a glyph with features belonging to this method are displayed in the viewer widget.

The "Upload features" tab

The data have to be provided in a tab separated text file format containing a header line, which describes the columns.

1. Select the csv file containing your annotation data. The first line in the have to column labels. The columns should be tab-separated.
2. Select the method which has produced the data. See previous section how to define a new method.
3. Assign the data to a certain genome.
4. Coordinates of the features can be provided in two way:.
   a) as **genomic** coordinates. Then, the chromosome, start and end position must be specified.
   b) **relativ** to the sequence start of the corresponding promoter sequence (specified by its gene ACCs).
5. Assign the corresponding columns between your file and *D-Light*.
6. Select the data rows you wish to upload and click "Send". Then following tasks are performed:
   - In case the coordinate type is "relativ", the gene ACCs in the sequence column are checked to exits in *D-Light*. If the ACCs are not in *D-Light* the corresponding the corresponding rows are skipped.
   - In case "genomic" coordinates are used, *D-Light* determines the promoter(s) which belong(s) to the given chromosome and intersects the given region. Then the feature is assigned. Lines with coordinates outside the stored promoter regions are ignored.
   - Feature ACCs which are not yet defined in *D-Light* are created as "general feature" without an associated PFM.

The "Upload feature" function is considered to upload in-house data with a maxium of a few thousands of features. At the moment as single file may not exceed 5 megabytes."

The "Upload Matrices" tab

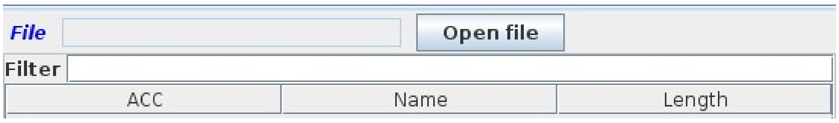

Uploads multiple PFMs from a file. The file format taken from JASPAR. Each record starts with a fasta style header line

```
>ACC NAME
```

follow by the counts for the four bases.

```
A  [ pos1  pos2 ...  posN ]
C  [ pos1  pos2 ...  posN ]
G  [ pos1  pos2 ...  posN ]
T  [ pos1  pos2 ...  posN ]
```

The characters A,C,G,T at the beginning of a line a and the brackets `[ ]` are mandatory. Here is an example with two matrices:

```
>MA0259.1 HIF1A::ARNT
A  [ 27  10  78   0   0   0   0  18 ]
C  [ 28  29   2 103   0   0   0  51 ]
G  [ 49  34  23   0 104   0 104  20 ]
T  [  0  31   1   1   0 104   0  15 ]
>MA0442.1 SOX10
A  [ 0  8  0  0  0  0 ]
C  [19  2  1  0  3  0 ]
G  [ 0  2  4  0 19  1 ]
T  [ 3 10 17 22  0 21 ]
```

The "Upload Sequences" tab

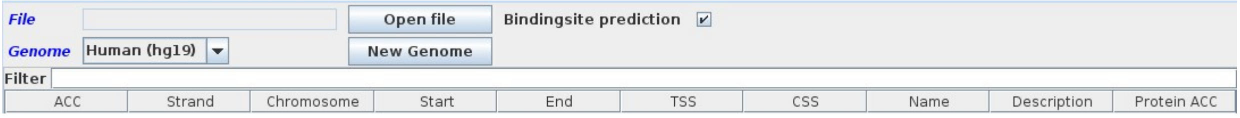

Here, a multi-fasta file can be uploaded for integration into *D-Light*. either into an existing genome, or in a freshly created on. In the second case press the "New Genome" button.

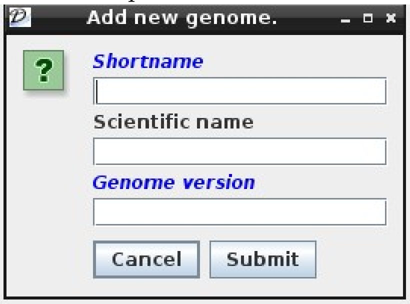

If, for example, you want to add the bovine genome, "Shortname" should be set to "Cow", "Scientific Name" to "Bos taurus" and "Genome Version" to "bosTau7".

UCSC derived promoter sequence files are available from our web pages:
`http://biwww.che.sbg.ac.at/dlight/files/promoters`

Once the sequences are stored and the "Binding site prediction" check box is checked, the PFM scanner is started and TFBS's are calculated for all stored PFMs. Since *D-Light* can make use of genomic coordinates, these coordinates can be supplied in the fasta header, together with other information. The header format then needs to be as follows, with fields separated by `|`.

```
>AccessionCode|strand|chromosome|start|end|TSS|CSS|ShortName|Description|ProteinAcc
```

e.g.:

```
>NM_001034679.1|f|chr1|350708|352707|351707|351787|RCAN1|calcipressin-1|NP_001029851
```

As soon as *D-Light* detects *one* vertical bar | , all are assumed to be existent **but** enclosed fields may be empty.

e.g.: no coordinates but short name and description:
`>NM_001034679.1||||||RCAN1|calcipressin-1|`

or: coordinates but not TSS and CSS:
`>NM_001034679.1|f|chr1|350708|352707|||RCAN1|calcipressin-1|NP_001029851`

If no | appears, the first word after the "greater than" sign > is taken as the accession code. The following two lines have the same meaning:

```
>NM_001034679.1|||||||||
>NM_001034679.1
```

The "Upload X-ref" tab

Homology relations can be defined by the user in csv file and uploaded to *D-Light*. The csv file requires a header line with the genome version name (hg19, mm10, etc.) Then, one line per homologous group of genes need to be given by the corresponding accession codes. If in one of the given genomes there are no homologs (known), put a - sign there. Paralogs should be separated by commas. E.g.:

```
rn4 mm10 hg19 bosTau6
NM_001126092.2  NM_175102.4     NM_031287.2     NM_001025350.2
NM_001106217.1 NM_172784.3 NM_032832.5 -
NM_001008962.1,NM_001008961.1,NM_001008960.1,NM_001008958.1     NM_134196.1     -       -
...
```

Note that the current state of assignments can be downloaded via "Query→Datasets→Download Dataset→X-ref". Use this as a template, add your own assignments and upload the extended table (or the subset you have changed).

4 Server Installation

The server side software can reside at any place on your server host. The installation does not require root permissions per se. The *D-Light* server is listening to two distinct TCP ports (default 8080 for DAS and 1099 for Java RMI). The web server part is only responsible for offering the client software and does not communicate to the *D-Light* server. Installation of the web server components may require root permissions. Also, if you want the *D-Light* server to be started automatically after system reboot, root permissions are required to place the start/stop script in /etc/init.d .

4.1 System Requirements

4.1.1 Hardware

- Disk-space estimate for the default ±2000 BP promoter data set for Human + Mouse + Rat: 2 GB for the basic installation. Be aware that users can add considerable amounts of new data. The space requirement grows linearly with the promoter sizes. We recommend to allocate a 5-10 GB for the *D-Light*database.
- 1GB RAM

4.1.2 OS Versions

We tested several x86 based Linux systems. But any UNIX like OS which offers the packages listed below should work.

Linux distributions known to work:

- openSUSE 12.1 (64 Bit)
- Ubuntu 12.04 LTS (64 Bit)
- DEBIAN squeeze (64 Bit)
- Fedora 17 (64 Bit)

Refer to the `README.PACKAGES` file for installation support for these systems. The file is part of the server distribution and located in the `dloprom-1.1` directory after unpacking.

4.1.3 Software

- cmake 2.6 or later (older version may also work)
- SWIG 1.3 (older version may also work)
- gcc/g++ 4.x (g++ 3.x and other C++ compilers supported by cmake also may work).
- Java **JDK** 1.6 or later (we used Sun Java JDK 1.6). Check that your Java installation contains the file `tools.jar` in `JAVA_HOME/lib/`. If not, your Java installation is not the JDK version or the installation is corrupt. Please install the current version of Java JDK, in this case (`http://java.sun.com/javase/downloads/index.jsp`). If you use a Linux package management ensure to have installed both, the Java runtime and the Java development package.
- MySQL 5.1 or later server (5.0 may also work)

- Python 2.5 or 2.6 (2.7 should work, Python 3 will not work)
- the python-mysql extension
- the python-devel extension
- wget

4.2 Server setup

The setup is done by Python scripts. Before you do anything else, check if python 2.5 or 2.6 is installed by typing: `python -V` .

The installation is separated in three steps:

- Installing the software components
- Installing the initial data sets
- Preparing the website (optional)

4.2.1 Software Installation

The software installation will take about 10-15 minutes.

(i) The *D-Light* server requires access to a MySQL server. The MySQL server does not need to be located on the *D-Light* server host. If you don't have access to a MySQL server, install one and be sure that it is up and running before you proceed.
(ii) The DAS server component (MyDas) of *D-Light* employs Maven which dynamically loads software components from the web during the *D-Light* server startup. Also the initial data setup loads sequence and TBFS data from the web. Therefore the *D-Light* server host must be connected to the internet and a potential firewall needs to allow outgoing connection for http and ftp services.

- unpack the tar file on the server host: `tar xvzf dloprom-1.1.tar.gz`
- change to the dloprom-1.1 directory
- run the installation script: `python server-setup.py`

The installation procedure checks for proper requirements and asks you to install some packages if required, using your favorite package manager (dpkg,apt,yum,YaST,Zypper,...).

During installation you will be prompted for an installation target directory. This directory will be called *DLOPROMBASE* below.

Furthermore the script will ask you for . . .

- a path to your JDK 1.6 installation, if `$JAVA_HOME` is not set or if the version of the default Java runtime is lower than 1.6
- the MySQL administrator's name and password.

- a D-Light database name.
- the name (and password) of a MySQL user, which should get full access to this database.
- the DNS-name or IP-address of the host machine.
- a free IP-Port for DAS-services.
- a free IP-Port for RMI-services.
- the maximum available memory for D-Light services (at least 1 GB).

After the installation *DLOPROMBASE* will contain the following subdirectories:

- `client`: contains the *D-Light*client and its configuration file
- `server`: contains the data and the server side implementation
- `web`: contains all files necessary for the website

Beside the subdirectories *DLOPROMBASE* will also contain two configuration files (`environment.config` and `dlight.properties`) and a start script (`start-server.py`). For starting the server change to *DLOPROMBASE* and run the start script `python dlightsrv.py start` . To stop the *D-Light* server use: `python dlightsrv.py stop` . To query the server status use `python dlightsrv.py status` .

We recommend to copy the provided `dlightsrv` init script from *DLOPROMBASE* to `/etc/init.d`. Create proper symlinks in `/etc/init.d/rc3.d` and `/etc/init.d/rc5.d`. Either as root or with sudo run e.g.:

```
cd /etc/init.d/r3.d
ln -s ../dlightsrv S07dlightsrv
ln -s ../dlightsrv K01dlightsrv
cd /etc/init.d/r5.d
ln -s ../dlightsrv S07dlightsrv
ln -s ../dlightsrv K01dlightsrv
```

If one of the parameters given during the installation, changes (IP-address, port . . .) or if you move *DLOPROMBASE* later on, you have to adapt the two configuration files.

4.2.2 Initial data setup

The initial data setup requires approx. 1-3 hours with default setting (1 hour for modern hardware, 3 hours for older desktop machines), as we calculate the annotation data during the setup procedure.

- change to `DLOPROMBASE/server/scripts` subdirectory, which is created during software setup
- load environment `DLOPROMBASE/environment.config` (`source ../../environment.config`)
- edit the file `setup.xml` to specify your data sets
- execute: `python fillDb.py <MYSQL-HOST> <DATABASE> <MYSQL-USER>`
  - the script takes three arguments:
    1. MYSQL-HOST: host of the MySQL database, which you have defined during the setup
    2. DATABASE: name of the MySQL database, which you have defined during the setup
    3. MYSQL-USER: user with insert, update, and create privileges to the MySQL database
  - example: `python fillDb.py localhost dlightDb dlight`

– the script calls the four scripts (init-dloprom.py, fillDb_Annotations.py, fillDb_Sequences.py, and run-scanner.py) to initialize the MySQL database.

this step may take several hours on a standard PC hardware for the very large datasets.

You can reset the dataset by execute the script resetDb.py (in DLOPROMBASE/server/scripts) Attention! This script removes all information, except usernames and passwords, from the database.

4.2.3 Prepare a *D-Light* website

This assumes that a web-server is up and running on the *D-Light* host. Note that the *D-Light* website is just responsible for providing the the *D-Light* client software. Root permission may be required to copy the *D-Light* files to the web servers documents tree.

Normally a Java applet runs in a sandbox on the client and has no access to resources of the clients operating system (e.g. printing, safe or load files…). The *D-Light* package provides functionality to save or print scan results locally, which needs access to system resources. This violates the rules of the sandbox.

For this reason, you have to sign the .jar files in the DLOPROMBASE/web directory, if you like to provide the applet client. If you don't like to do this, do not offer the *D-Light* web site and only provide the stand alone client to your users.

Once the jar files are signed you can copy them from DLOPROMBASE/web to your web directory (i.e. /srv/www/htdocs/dlight).

Java JDK provides tools (keytool and jarsigner)[1] to sign the files. In a first step you have to create a keystore:

```
keytool -genkey -keystore <storename> -alias <aliasName>
e.g.:
keytool -genkey -keystore dlightstore -alias "Bill"
```

After that, you can sign the jarfiles (DLight_GUI.jar and genoviz.jar):

```
jarsigner -keystore <storename> DLight_GUI.jar <aliasName>
jarsigner -keystore <storename> genoviz.jar <aliasName>
```

If you like to provide the Java webstart version of the *D-Light* client, you have to sign the jarfile DLight_Client.jar as well:

```
jarsigner -keystore <storename> DLight_Client.jar <aliasName>
```

For further information see:

- http://wiki.plexinfo.net/index.php?title=How_to_sign_JAR_files
- http://java.sun.com/developer/Books/javaprogramming/JAR/sign/signing.html

[1] Please make sure that you use the tools from a Java JDK 1.6 or later installation for this.
