# Supplementary material for: D-Light on promoters: a client-server system for the analysis and visualization of cis-regulatory elements
Source: BMC Bioinformatics. 2013 Apr 24;14:140. doi: 10.1186/1471-2105-14-140 (PMC3685601; doi:10.1186/1471-2105-14-140)
Supplement: Additional file 3 — Installation package. Server and client software for local installation. [file 1471-2105-14-140-S3.zip › dloprom-1.1/licenses/LICENSE_genoviz.html]

# Common Public License Version 1.0

THE ACCOMPANYING PROGRAM IS PROVIDED UNDER THE TERMS OF THIS COMMON PUBLIC
LICENSE ("AGREEMENT"). ANY USE, REPRODUCTION OR DISTRIBUTION OF THE PROGRAM
CONSTITUTES RECIPIENT'S ACCEPTANCE OF THIS AGREEMENT.

**1. DEFINITIONS**

"Contribution" means:

> a) in the case of the initial Contributor, the initial code and documentation
> distributed under this Agreement, and
>
> b) in the case of each subsequent Contributor:
>
> i) changes to the Program, and
>
> ii) additions to the Program;
>
> where such changes and/or additions to the Program originate from and are
> distributed by that particular Contributor. A Contribution 'originates' from a
> Contributor if it was added to the Program by such Contributor itself or anyone
> acting on such Contributor's behalf. Contributions do not include additions to
> the Program which: (i) are separate modules of software distributed in
> conjunction with the Program under their own license agreement, and (ii) are
> not derivative works of the Program.

"Contributor" means any person or entity that distributes the Program.

"Licensed Patents " mean patent claims licensable by a Contributor which are
necessarily infringed by the use or sale of its Contribution alone or when
combined with the Program.

"Program" means the Contributions distributed in accordance with this
Agreement.

"Recipient" means anyone who receives the Program under this Agreement,
including all Contributors.

**2. GRANT OF RIGHTS**

> a) Subject to the terms of this Agreement, each Contributor hereby grants
> Recipient a non-exclusive, worldwide, royalty-free copyright license to
> reproduce, prepare derivative works of, publicly display, publicly perform,
> distribute and sublicense the Contribution of such Contributor, if any, and
> such derivative works, in source code and object code form.
>
> b) Subject to the terms of this Agreement, each Contributor hereby grants
> Recipient a non-exclusive, worldwide, royalty-free patent license under
> Licensed Patents to make, use, sell, offer to sell, import and otherwise
> transfer the Contribution of such Contributor, if any, in source code and
> object code form. This patent license shall apply to the combination of the
> Contribution and the Program if, at the time the Contribution is added by the
> Contributor, such addition of the Contribution causes such combination to be
> covered by the Licensed Patents. The patent license shall not apply to any
> other combinations which include the Contribution. No hardware per se is
> licensed hereunder.
>
> c) Recipient understands that although each Contributor grants the licenses
> to its Contributions set forth herein, no assurances are provided by any
> Contributor that the Program does not infringe the patent or other intellectual
> property rights of any other entity. Each Contributor disclaims any liability
> to Recipient for claims brought by any other entity based on infringement of
> intellectual property rights or otherwise. As a condition to exercising the
> rights and licenses granted hereunder, each Recipient hereby assumes sole
> responsibility to secure any other intellectual property rights needed, if any.
> For example, if a third party patent license is required to allow Recipient to
> distribute the Program, it is Recipient's responsibility to acquire that
> license before distributing the Program.
>
> d) Each Contributor represents that to its knowledge it has sufficient
> copyright rights in its Contribution, if any, to grant the copyright license
> set forth in this Agreement.

**3. REQUIREMENTS**

A Contributor may choose to distribute the Program in object code form under
its own license agreement, provided that:

> a) it complies with the terms and conditions of this Agreement; and
>
> b) its license agreement:
>
> i) effectively disclaims on behalf of all Contributors all warranties and
> conditions, express and implied, including warranties or conditions of title
> and non-infringement, and implied warranties or conditions of merchantability
> and fitness for a particular purpose;
>
> ii) effectively excludes on behalf of all Contributors all liability for
> damages, including direct, indirect, special, incidental and consequential
> damages, such as lost profits;
>
> iii) states that any provisions which differ from this Agreement are offered
> by that Contributor alone and not by any other party; and
>
> iv) states that source code for the Program is available from such
> Contributor, and informs licensees how to obtain it in a reasonable manner on
> or through a medium customarily used for software exchange.

When the Program is made available in source code form:

> a) it must be made available under this Agreement; and
>
> b) a copy of this Agreement must be included with each copy of the Program.

Contributors may not remove or alter any copyright notices contained within
the Program.

Each Contributor must identify itself as the originator of its Contribution,
if any, in a manner that reasonably allows subsequent Recipients to identify
the originator of the Contribution.

**4. COMMERCIAL DISTRIBUTION**

Commercial distributors of software may accept certain responsibilities with
respect to end users, business partners and the like. While this license is
intended to facilitate the commercial use of the Program, the Contributor who
includes the Program in a commercial product offering should do so in a manner
which does not create potential liability for other Contributors. Therefore, if
a Contributor includes the Program in a commercial product offering, such
Contributor ("Commercial Contributor") hereby agrees to defend and indemnify
every other Contributor ("Indemnified Contributor") against any losses, damages
and costs (collectively "Losses") arising from claims, lawsuits and other legal
actions brought by a third party against the Indemnified Contributor to the
extent caused by the acts or omissions of such Commercial Contributor in
connection with its distribution of the Program in a commercial product
offering. The obligations in this section do not apply to any claims or Losses
relating to any actual or alleged intellectual property infringement. In order
to qualify, an Indemnified Contributor must: a) promptly notify the Commercial
Contributor in writing of such claim, and b) allow the Commercial Contributor
to control, and cooperate with the Commercial Contributor in, the defense and
any related settlement negotiations. The Indemnified Contributor may
participate in any such claim at its own expense.

For example, a Contributor might include the Program in a commercial product
offering, Product X. That Contributor is then a Commercial Contributor. If that
Commercial Contributor then makes performance claims, or offers warranties
related to Product X, those performance claims and warranties are such
Commercial Contributor's responsibility alone. Under this section, the
Commercial Contributor would have to defend claims against the other
Contributors related to those performance claims and warranties, and if a court
requires any other Contributor to pay any damages as a result, the Commercial
Contributor must pay those damages.

**5. NO WARRANTY**

EXCEPT AS EXPRESSLY SET FORTH IN THIS AGREEMENT, THE PROGRAM IS PROVIDED ON AN
"AS IS" BASIS, WITHOUT WARRANTIES OR CONDITIONS OF ANY KIND, EITHER EXPRESS OR
IMPLIED INCLUDING, WITHOUT LIMITATION, ANY WARRANTIES OR CONDITIONS OF TITLE,
NON-INFRINGEMENT, MERCHANTABILITY OR FITNESS FOR A PARTICULAR PURPOSE. Each
Recipient is solely responsible for determining the appropriateness of using
and distributing the Program and assumes all risks associated with its exercise
of rights under this Agreement, including but not limited to the risks and
costs of program errors, compliance with applicable laws, damage to or loss of
data, programs or equipment, and unavailability or interruption of operations.

**6. DISCLAIMER OF LIABILITY**

EXCEPT AS EXPRESSLY SET FORTH IN THIS AGREEMENT, NEITHER RECIPIENT NOR ANY
CONTRIBUTORS SHALL HAVE ANY LIABILITY FOR ANY DIRECT, INDIRECT, INCIDENTAL,
SPECIAL, EXEMPLARY, OR CONSEQUENTIAL DAMAGES (INCLUDING WITHOUT LIMITATION LOST
PROFITS), HOWEVER CAUSED AND ON ANY THEORY OF LIABILITY, WHETHER IN CONTRACT,
STRICT LIABILITY, OR TORT (INCLUDING NEGLIGENCE OR OTHERWISE) ARISING IN ANY
WAY OUT OF THE USE OR DISTRIBUTION OF THE PROGRAM OR THE EXERCISE OF ANY RIGHTS
GRANTED HEREUNDER, EVEN IF ADVISED OF THE POSSIBILITY OF SUCH DAMAGES.

**7. GENERAL**

If any provision of this Agreement is invalid or unenforceable under
applicable law, it shall not affect the validity or enforceability of the
remainder of the terms of this Agreement, and without further action by the
parties hereto, such provision shall be reformed to the minimum extent
necessary to make such provision valid and enforceable.

If Recipient institutes patent litigation against a Contributor with respect
to a patent applicable to software (including a cross-claim or counterclaim in
a lawsuit), then any patent licenses granted by that Contributor to such
Recipient under this Agreement shall terminate as of the date such litigation
is filed. In addition, if Recipient institutes patent litigation against any
entity (including a cross-claim or counterclaim in a lawsuit) alleging that the
Program itself (excluding combinations of the Program with other software or
hardware) infringes such Recipient's patent(s), then such Recipient's rights
granted under Section 2(b) shall terminate as of the date such litigation is
filed.

All Recipient's rights under this Agreement shall terminate if it fails to
comply with any of the material terms or conditions of this Agreement and does
not cure such failure in a reasonable period of time after becoming aware of
such noncompliance. If all Recipient's rights under this Agreement terminate,
Recipient agrees to cease use and distribution of the Program as soon as
reasonably practicable. However, Recipient's obligations under this Agreement
and any licenses granted by Recipient relating to the Program shall continue
and survive.

Everyone is permitted to copy and distribute copies of this Agreement, but in
order to avoid inconsistency the Agreement is copyrighted and may only be
modified in the following manner. The Agreement Steward reserves the right to
publish new versions (including revisions) of this Agreement from time to time.
No one other than the Agreement Steward has the right to modify this Agreement.
IBM is the initial Agreement Steward. IBM may assign the responsibility to
serve as the Agreement Steward to a suitable separate entity. Each new version
of the Agreement will be given a distinguishing version number. The Program
(including Contributions) may always be distributed subject to the version of
the Agreement under which it was received. In addition, after a new version of
the Agreement is published, Contributor may elect to distribute the Program
(including its Contributions) under the new version. Except as expressly stated
in Sections 2(a) and 2(b) above, Recipient receives no rights or licenses to
the intellectual property of any Contributor under this Agreement, whether
expressly, by implication, estoppel or otherwise. All rights in the Program not
expressly granted under this Agreement are reserved.

This Agreement is governed by the laws of the State of New York and the
intellectual property laws of the United States of America. No party to this
Agreement will bring a legal action under this Agreement more than one year
after the cause of action arose. Each party waives its rights to a jury trial
in any resulting litigation.
