# Supplementary material for: D-Light on promoters: a client-server system for the analysis and visualization of cis-regulatory elements
Source: BMC Bioinformatics. 2013 Apr 24;14:140. doi: 10.1186/1471-2105-14-140 (PMC3685601; doi:10.1186/1471-2105-14-140)
Supplement: Additional file 3 — Installation package. Server and client software for local installation. [file 1471-2105-14-140-S3.zip › dloprom-1.1/install/web/index.html]

D-Light navigation


|  | --- |  |
| **Java Applet** | **Java Webstart** | **Standalone Client (download)** |
|  | --- |  |
|  | **Manual** |
|  | Administration Contact: admin@x.y.z |

|  |  |
| --- | --- |
| © D-Light Team, University of Salzburg / Upper Austria University of Applied Science, 2010-2011   D-Light on Promoters is free software and licensed under GNU General Public License, version 3. | |
