# Supplementary figures and images for: D-Light on promoters: a client-server system for the analysis and visualization of cis-regulatory elements
Source: BMC Bioinformatics. 2013 Apr 24;14:140. doi: 10.1186/1471-2105-14-140 (PMC3685601; doi:10.1186/1471-2105-14-140)

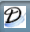

Supplement: Additional file 3 — Installation package. Server and client software for local installation. [file 1471-2105-14-140-S3.zip › dloprom-1.1/install/client/icon.png]

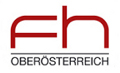

Supplement: Additional file 3 — Installation package. Server and client software for local installation. [file 1471-2105-14-140-S3.zip › dloprom-1.1/install/web/LogoFH2.jpg]

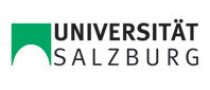

Supplement: Additional file 3 — Installation package. Server and client software for local installation. [file 1471-2105-14-140-S3.zip › dloprom-1.1/install/web/LogoUniSalzburg.jpg]

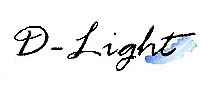

Supplement: Additional file 3 — Installation package. Server and client software for local installation. [file 1471-2105-14-140-S3.zip › dloprom-1.1/install/web/DlightLogo.jpg]

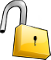

Supplement: Additional file 4 — Source code package. Source code of server and client. [file 1471-2105-14-140-S4.zip › dloprom-1.1-src/client_and_rmi/src/at/fh_hagenberg/dlight/threads/openPadlock.png]

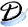

Supplement: Additional file 4 — Source code package. Source code of server and client. [file 1471-2105-14-140-S4.zip › dloprom-1.1-src/client_and_rmi/src/at/fh_hagenberg/dlight/gui/logo.png]

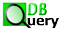

Supplement: Additional file 4 — Source code package. Source code of server and client. [file 1471-2105-14-140-S4.zip › dloprom-1.1-src/client_and_rmi/src/at/fh_hagenberg/dlight/gui/panels/query.png]

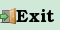

Supplement: Additional file 4 — Source code package. Source code of server and client. [file 1471-2105-14-140-S4.zip › dloprom-1.1-src/client_and_rmi/src/at/fh_hagenberg/dlight/gui/panels/LogoutSmall.png]

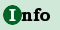

Supplement: Additional file 4 — Source code package. Source code of server and client. [file 1471-2105-14-140-S4.zip › dloprom-1.1-src/client_and_rmi/src/at/fh_hagenberg/dlight/gui/panels/ViewPromoterSmall.png]

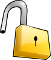

Supplement: Additional file 4 — Source code package. Source code of server and client. [file 1471-2105-14-140-S4.zip › dloprom-1.1-src/client_and_rmi/src/at/fh_hagenberg/dlight/gui/panels/openPadlock.png]

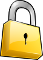

Supplement: Additional file 4 — Source code package. Source code of server and client. [file 1471-2105-14-140-S4.zip › dloprom-1.1-src/client_and_rmi/src/at/fh_hagenberg/dlight/gui/panels/closedPadlock.png]

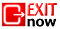

Supplement: Additional file 4 — Source code package. Source code of server and client. [file 1471-2105-14-140-S4.zip › dloprom-1.1-src/client_and_rmi/src/at/fh_hagenberg/dlight/gui/panels/exit.png]

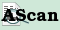

Supplement: Additional file 4 — Source code package. Source code of server and client. [file 1471-2105-14-140-S4.zip › dloprom-1.1-src/client_and_rmi/src/at/fh_hagenberg/dlight/gui/panels/ScanAnnotationSmall.png]

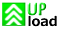

Supplement: Additional file 4 — Source code package. Source code of server and client. [file 1471-2105-14-140-S4.zip › dloprom-1.1-src/client_and_rmi/src/at/fh_hagenberg/dlight/gui/panels/upload.png]

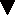

Supplement: Additional file 4 — Source code package. Source code of server and client. [file 1471-2105-14-140-S4.zip › dloprom-1.1-src/client_and_rmi/src/at/fh_hagenberg/dlight/gui/panels/selPopup.png]

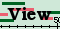

Supplement: Additional file 4 — Source code package. Source code of server and client. [file 1471-2105-14-140-S4.zip › dloprom-1.1-src/client_and_rmi/src/at/fh_hagenberg/dlight/gui/panels/ViewScanSmall.png]

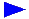

Supplement: Additional file 4 — Source code package. Source code of server and client. [file 1471-2105-14-140-S4.zip › dloprom-1.1-src/client_and_rmi/src/at/fh_hagenberg/dlight/gui/panels/PfeilBlau.gif]

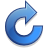

Supplement: Additional file 4 — Source code package. Source code of server and client. [file 1471-2105-14-140-S4.zip › dloprom-1.1-src/client_and_rmi/src/at/fh_hagenberg/dlight/gui/refresh.png]
