# Supplementary material for: D-Light on promoters: a client-server system for the analysis and visualization of cis-regulatory elements
Source: BMC Bioinformatics. 2013 Apr 24;14:140. doi: 10.1186/1471-2105-14-140 (PMC3685601; doi:10.1186/1471-2105-14-140)
Supplement: Additional file 3 — Installation package. Server and client software for local installation. [file 1471-2105-14-140-S3.zip › dloprom-1.1/install/das/dlightDAS.tar.gz!/dlightDAS/src/main/webapp/xslt/dsn-simple.html]

Mydas: Data Source


mydas

### Data Sources

| Short Name | Full Name | Version | Mapmaster | Description |
| --- | --- | --- | --- | --- |
| ipi-tryptic | International Protein Index Predicted Tryptic Peptides | 0.1 | http://www.ebi.ac.uk/tc-test/das-srv/uniprot/das/ipi | Theoretical tryptic peptides based upon a complete and successful digest of the requested proteins with trypsin. |
| uniprot-tryptic | UniProt Predicted Tryptic Peptides | 0.1 | http://www.ebi.ac.uk/tc-test/das-srv/uniprot/das/uniprot | Theoretical tryptic peptides based upon a complete and successful digest of the requested proteins with trypsin. |

European Bioinformatics Institute © 2007. All rights reserved.
