# Supplementary material for: D-Light on promoters: a client-server system for the analysis and visualization of cis-regulatory elements
Source: BMC Bioinformatics. 2013 Apr 24;14:140. doi: 10.1186/1471-2105-14-140 (PMC3685601; doi:10.1186/1471-2105-14-140)
Supplement: Additional file 3 — Installation package. Server and client software for local installation. [file 1471-2105-14-140-S3.zip › dloprom-1.1/install/das/dlightDAS.tar.gz!/dlightDAS/src/main/webapp/xslt/features.html]

MyDAS: Data Sources


mydas

### Summary [hide]

|  |  |
| --- | --- |
| ID | P12345 |
| Description | Sample protein |
| Length | 123 |

### Positional features [hide]

| Category | Type | Feature |  |
| --- | --- | --- | --- |
| Molecule Processing | HELIX | BRCA1\_HUMAN\_HELIX\_3\_50 |  |
| ignore | STRAND | BRCA1\_HUMAN\_STRAND\_52\_70 |  |

### Non-positional features [hide]

| Source | Type | Feature | Method | Score | Notes |
| --- | --- | --- | --- | --- | --- |
| uniprot | reference | pubmed:9760198 | reference | - | Dong J., Chang-Claude J., Wu Y., Schumacher V., Debatin I., Tonin P., Royer-Pokora B. (1998). "A high proportion of mutations in the BRCA1 gene in German breast/ovarian cancer families with clustering of mutations in the 3' third of the gene.", Hum. Genet. 103:154-161 |
| ignore | reference | pubmed:10500182 | reference | 3.3 | Lorick K.L., Jensen J.P., Fang S., Ong A.M., Hatakeyama S., Weissman A.M. (1999). "RING fingers mediate ubiquitin-conjugating enzyme (E2)-dependent ubiquitination.", Proc. Natl. Acad. Sci. U.S.A. 96:11364-11369 |

European Bioinformatics Institute © 2007. All rights reserved.
